# State-space models’ dirty little secrets: even simple linear Gaussian models can have estimation problems

**DOI:** 10.1038/srep26677

**Published:** 2016-05-25

**Authors:** Marie Auger-Méthé, Chris Field, Christoffer M. Albertsen, Andrew E. Derocher, Mark A. Lewis, Ian D. Jonsen, Joanna Mills Flemming

**Affiliations:** 1 Dalhousie University, Department of Mathematics and Statistics, Halifax, B3H 4R2, Canada; 2 Technical University of Denmark, National Institute of Aquatic Resources, Charlottenlund, 2920, Denmark; 3 University of Alberta, Department of Biological Sciences, Edmonton, T6G 2E9, Canada; 4 University of Alberta, Department of Mathematical and Statistical Sciences, Edmonton, T6G 2G1, Canada; 5 Macquarie University, Department of Biological Sciences, Sydney, 2109, Australia

## Abstract

State-space models (SSMs) are increasingly used in ecology to model time-series such as animal movement paths and population dynamics. This type of hierarchical model is often structured to account for two levels of variability: biological stochasticity and measurement error. SSMs are flexible. They can model linear and nonlinear processes using a variety of statistical distributions. Recent ecological SSMs are often complex, with a large number of parameters to estimate. Through a simulation study, we show that even simple linear Gaussian SSMs can suffer from parameter- and state-estimation problems. We demonstrate that these problems occur primarily when measurement error is larger than biological stochasticity, the condition that often drives ecologists to use SSMs. Using an animal movement example, we show how these estimation problems can affect ecological inference. Biased parameter estimates of a SSM describing the movement of polar bears (*Ursus maritimus*) result in overestimating their energy expenditure. We suggest potential solutions, but show that it often remains difficult to estimate parameters. While SSMs are powerful tools, they can give misleading results and we urge ecologists to assess whether the parameters can be estimated accurately before drawing ecological conclusions from their results.

State-space models (SSMs) are increasingly used in ecology and are becoming the favoured statistical framework for modelling animal movement and population dynamics^1^,^2^,^3^,^4^. SSMs are desirable because they are structured so as to differentiate between two distinct sources of variability: the biological or process variation (e.g., demographic stochasticity) and the measurement error associated with the sampling method^2^,^4^. Because marine observations are often associated with large measurement errors that can mask biological signals, much of the early development of SSMs in ecology was by marine ecologists and fisheries scientists (e.g.^5^,^6^,^7^). The SSM framework has since become a general approach to account for multiple levels of stochasticity when modelling time-series, making them increasingly popular in the terrestrial literature (e.g.^8^,^9^,^10^). Here, we demonstrate that even simple SSMs can be problematic. The model we chose is often used to explain how SSMs can account for two levels of stochasticity (e.g.^4^), yet, we show that it suffers from parameter- and state-estimation problems.

SSMs are a type of hierarchical model, in which one level treats the underlying unobserved states as an autocorrelated process, while another level accounts for measurement error^11^. The SSM framework is flexible, especially when fitted with Monte Carlo methods such as particle filters or Markov Chain Monte Carlo (MCMC). SSMs can be used to model a variety of linear and nonlinear processes, and can represent stochasticity with diverse statistical distributions (e.g.^3^,^12^,^13^). The flexibility of the SSM approach allows ecologists to build complex models that describe the biological and measurement processes with levels of detail that were previously unattainable.

While the SSM framework is flexible, much of its theoretical foundation is based on simple linear Gaussian SSMs (sometimes referred as normal dynamic linear models, see Newman *et al.*^4^). An example of a simple univariate linear Gaussian SSM is the one we will use to demonstrate parameter-estimability problems:


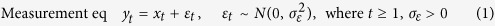



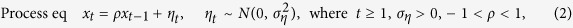


where 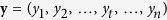 are observed at regular time intervals 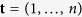 for a time-series of length *n* and 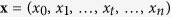 are the true unobserved states, with *x*_0_ representing the initial state. An ecological example of such a time-series would be a series of yearly population size estimates. For instance, Newman *et al.*^4^ use this model to introduce SSM for population dynamics with *x*_*t*_ representing the true but unknown abundance of an animal population at time *t*, *y*_*t*_ an unbiased observation of the population size at time *t*, and *ρ* the population growth rate.

The origin of SSMs is intimately linked with the Kalman filter, a recursive procedure to estimate the unobserved states based on inaccurate observations (e.g., estimating the true fish abundance based on catch data). The Kalman filter was developed to estimate states based on a model without unknown parameter values^14^. However, in ecological applications, most parameters need to be estimated (e.g.^3^). Fitting methods for SSMs, such as the Kalman filter, are now used to facilitate both state and parameter estimation^15^. In many cases, SSMs are used to estimate variance parameters because they are designed to differentiate measurement error from process stochasticity^16^,^17^. While estimating parameters is often a means to estimate the unobserved states (e.g.^13^,^15^), parameters themselves can be of interest because they describe the underlying dynamics of the system, or behaviour of the animal (e.g.^3^,^18^).

Estimability problems associated with SSMs and other hierarchical models have been discussed in the population dynamics literature (e.g.^16^,^19^). In particular, previous studies have emphasized how difficult it is to use SSMs to estimate density dependence parameters^19^,^20^ and to differentiate process stochasticity from measurement error (e.g.^16^). However, the existence of parameter estimation problems have been largely overlooked in the movement literature, and by those that use complex Bayesian SSMs. As SMMs are becoming the favoured framework for many ecological analyses^1^,^2^,^3^,^4^, and are gaining popularity in other fields (e.g.^21^), it is timely to warn researchers of their weaknesses.

Here, we use simulations to show that simple SSMs can have severe parameter-estimability problems that in turn affect state estimates. These problems are more frequent when the measurement error is large, the very condition under which SSMs are needed, and can persist even when we incorporate measurement error information. While our main estimation approach consists of maximizing the likelihood numerically through Template Model Builder (TMB)^22^, we show that these problems persist across a wide range of platforms and statistical frameworks, including when the parameters and states are estimated via Bayesian methods. We use the polar bear (*Ursus maritimus*) movement data that led us to notice these problems to demonstrate the effect of estimation problems on the biological interpretation of results. Finally, we discuss techniques to diagnose and, when possible, alleviate estimability problems.

## Methods

### Demonstration of the problem

When we fit models to data, we want the parameters to be identifiable, which means that, given perfect data (e.g., an infinitely long time-series), it is possible to learn the true values of parameters. Assessing parameter identifiability is often difficult and a more attainable goal is to assess estimability. Estimability means that, given the data at hand, the method used to approximate the parameter yields a unique estimate. When the maximum value of the likelihood function occurs at more than one parameter value, the parameter is nonestimable. The quality of parameter estimates can be assessed in terms of: its variance, measured over multiple repeated estimations; bias, the expected difference between the estimate and true value of the parameter; or mean square error, a composite of bias and variance. To demonstrate that the estimates of the parameters and states of SSMs can be inaccurate, we simulated a set of time-series using the model presented in equations 1, 2. In all simulations, the values for the initial state, *x*_0_, the measurement error, *σ*_*ε*_, and the correlation, *ρ*, were set to 0, 0.1, and 0.7, respectively. In Appendix A (Supplementary information), we explored other *ρ* values, including a simpler model where *ρ* is fixed to 1. Note that while this simpler model has fewer parameters to estimate, it is no longer stationary^23^. To investigate whether the ratio of measurement to process stochasticity affected estimation, we simulated a range of *σ*_*η*_ values: (0.01, 0.02, 0.05, 0.1, 0.2, 0.5, 1). For each parameter set, we simulated 200 time-series each with 100 observations (*n* = 100). Analyses using longer time-series (n = 500) are presented in Appendix B (Supplementary information).

For each simulation, we estimated the parameters, ***θ*** = (*σ*_*ε*_, *ρ*, *σ*_*η*_), and states, **x**, using the R^24^ package TMB. This R package is similar to AD Model Builder^25^ in that it uses automatic differentiation and the Laplace approximation. Finding the Maximum Likelihood Estimate (MLE) of the parameters of a SSM requires the maximization of the marginal distribution of the observations^4^. For the model presented in equations 1, 2, this involves maximizing the following likelihood:


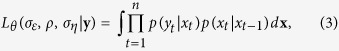



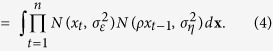


To get the marginal distribution, we integrate over the states, 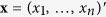. In TMB, this integration is achieved using the Laplace approximation, which in turn also returns state estimates^13^. While we refer to state “estimation”, this process is sometimes called “prediction” because states can be interpreted as random variables^4^. In this example, we assumed that the initial state is known (i.e., *x*_0_ = 0), which should help the estimation process. In instances where the initial state value is unavailable, the initial state can be modelled as *x*_0_ ~ *N*(*μ*, *σ*_0_)^23^. TMB calculates standard errors for the estimated parameters by using the inverse of the observed Fisher information, i.e. the Hessian of the log likelihood (similar to ADMB, see Fournier *et al.*^25^). To calculate the 95% confidence intervals (CI), we multiplied the aforementioned standard errors by the 2.5 and 97.5^th^ percentiles of the normal distribution (i.e., the quadratic approximation in Bolker^26^).

To demonstrate that the problem is widespread across different statistical platforms, we also fitted the simulated data using two popular R packages: dlm^27^ and rjags^28^. dlm uses the Kalman filter for the state estimates and calculates the MLE with numerical optimization methods. rjags is an R interface to JAGS^29^, a program that can be used to fit Bayesian hierarchical models using MCMC methods (Supplementary information: Appendix C).

We evaluated the parameter-estimation performance of SSMs by comparing the estimated and simulated values. Similar to Pedersen *et al.*^12^, we evaluated the state-estimation performance with the root mean square error (RMSE):


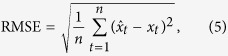


where 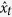 is the estimated state at time *t* and *x*_*t*_ is the simulated (i.e., true) state at time *t*. To assess whether the state-estimation performance was affected by the parameter-estimation problems, we compare 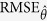, for which the parameters, ***θ*** = (*σ*_*ε*_, *ρ*, *σ*_*η*_), were also estimated, to RMSE_*θ*_, for which the parameter values were fixed at the values used to simulate the data.

To investigate the potential causes of the parameter-estimation problem, we explored the likelihood profile for a subset of the problematic simulations. We used the same simulations and parameter values as above, with the exception that we only examined the most problematic values: *σ*_*η*_ = (0.01, 0.02, 0.05) (see Results). Because they are associated with high measurement error to process stochasticity ratios, these values also represent the conditions when SSMs are most needed. For each scenario (i.e., different values of *σ*_*η*_), we randomly chose one simulation for which the 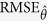 was 50% larger than RMSE_*θ*_. Again, we used TMB to estimate parameter values, ***θ***, and the states, **x**. To examine whether the estimation problems were associated with the simultaneous estimation of states and parameters, we estimated parameters when the state values were fixed to their simulated values (Supplementary information: Appendix D). As a final investigation of the causes of the estimation problems, we show how these problems are associated with known limitations of the autoregressive-moving-average (ARMA) models (Supplementary information: Appendix E).

### Incorporating measurement error information

Many ecologists incorporate information on measurement error in their model by either fixing parameter values or, in a Bayesian framework, using informative priors (e.g.^6^,^15^,^30^). We investigated whether fixing the measurement error resolved the parameter estimation problem. To do so, we fitted our simple likelihood (equation 4) to the same simulations, but we fixed the standard deviation of the measurement equation to the value used to simulate the data, *σ*_*ε*_ = 0.1. We only estimated the remaining parameters, 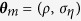. As above, we investigated the parameter estimates, RMSE of the states, and likelihood profiles.

### Ecological example

The movement of many animals, such as birds, fish and marine mammals, is a combination of the voluntary movement of the animal (active movement) and drift (passive displacement resulting from ocean or wind currents). Currents do not always direct animals towards their goals, and moving against currents may require a substantial amount of energy (e.g.^31^). To understand how currents affect the behavioural strategies of an animal, it is necessary to distinguish between the voluntary movement of the animal and drift^32^. The voluntary movement can then be used as a proxy of energy expenditure, or can be integrated into an energy budget model to assess the effects of movement on survival and reproduction^32^,^33^. While developments in satellite telemetry are providing increasingly precise measurements of animal movement paths, it is difficult to differentiate between drift and voluntary movement because wind, ocean, and sea ice drift data are often associated with large errors (e.g.^34^,^35^).

We noticed the estimation problems of linear Gaussian SSMs when developing a model that would differentiate between the voluntary movement of polar bears and sea ice drift. Polar bears often move in the reverse direction of the sea ice drift^36^,^37^ and sea ice drift can be associated with large errors^34^. As a proxy of energy expended by bears, we wanted to estimate the voluntary movement. As a first test, we developed a 2 dimensional SSM that accounts for error in ice drift data:


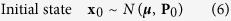



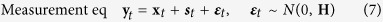



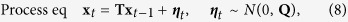


where 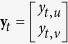 is the measured daily displacement of the polar bear based on the GPS collar data, 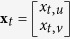 is the voluntary displacement of the polar bear, and 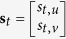 is the daily sea ice drift experienced by the bear. Here, the measurement error, ***ε***_*t*_, is associated with the ice data, not the polar bear location data. The location data were determined by GPS, for which the error is negligible (<30 *m*)^38^. For simplicity, we assumed that the two geographic coordinates are independent, thus:


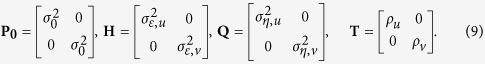


Because equations 6, 7, 8 model displacements, the elements of **H** represent the measurement error in the sea ice drift data and those of **Q** are associated with the speed of the bear. Similar to *γ* in Jonsen *et al.*^30^, *ρ*_*u*_ and *ρ*_*v*_ represent the degree of autocorrelation in the random walk. To initialize the model we used 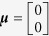 and 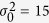. We chose 15 km as it is the standard deviation of the observed daily displacements of the polar bears in the *u*- and *v*-direction.

We used the daily movement of 15 polar bears collared in the Beaufort Sea in the spring of 2007–2011. The bears were immobilized with standard methods^39^ and equipped with Telonics Inc. (Mesa, AZ) collars. All capture and handling procedures were carried out in accordance with the protocols approved by the University of Alberta Animal Care and Use Committee for Biosciences. We used the Polar Pathfinder Daily 25 km Ease-Grid Sea Ice Motion Vectors^40^, which are daily estimates of sea ice displacements in the *u*- and *v*-directions of the Northern Hemisphere azimuthal equal-area EASE-Grid projection developed for polar sea ice data^41^. We used the same movement data and data handling procedures as in Auger-Méthé *et al.*^37^, including interpolating the ice drift data at each bear location, assigning a drift value of zero for landfast ice, and excluding the three days after collaring to remove movements affected by handling. The only differences in the data used here, are that we excluded all bears that spent time on land and considered days with missing sea ice data as missing observations (i.e., we considered both **y**_*t*_ and **s**_*t*_ as missing that day).

Our goal was to use the SSM to estimate the energy expenditure of each bear. Our proxy was the total voluntary bear displacement:


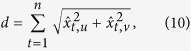


where 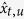 and 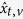 are the estimates of the daily voluntary bear displacements in the *u*- and *v*-directions. The number of days, *n*, included in the time-series will affect our estimate of *d*. For consistency, we set *n* to be 342, the length of the shortest time-series across the 15 bears. To assess the effects of estimation problems on our ecological interpretation, we simulated movement paths similar to those described by the polar bear data (Supplementary information: Appendix F).

The code is available at https://gitlab.oceantrack.org/otn-statistical-modelling-group/SSMestProblems and as Supplementary data.

## Results

### Simulations results

**Figure 1:**
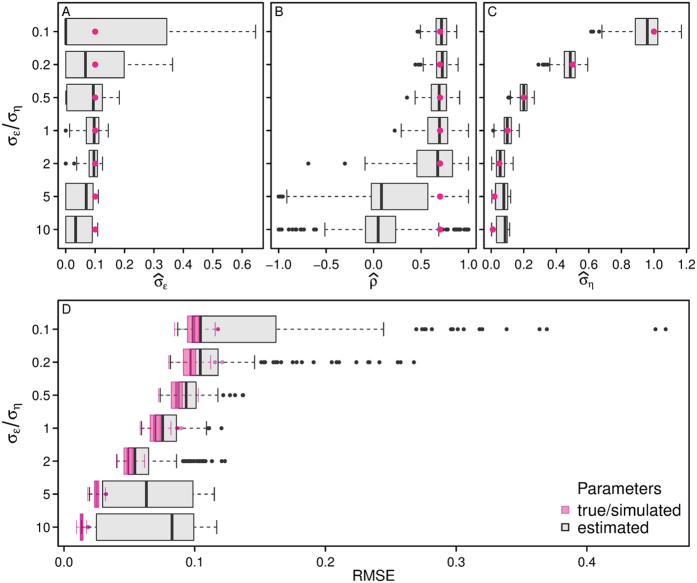
Changes in parameter estimates and state RMSEs associated with varying the measurement error to process stochasticity ratios (*σ*_*ε*_/*σ*_*η*_) in the simulations. (**A–C**) The boxplots represent the distribution of the parameter estimates (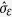, 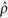, 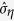) and the pink circles represent the true (simulated) values. (**D**) The grey boxplots represent the distribution of the RMSE of the model fitted using the estimated parameter values, while the pink boxplots represent the RMSE when the model is fitted using the true parameter values.

According to the simulation results, parameter estimation was often inaccurate, and these problems affected the state estimates (Fig. 1). The parameter estimates were often far from their true values, and their distributions often bimodal (Fig. 1, Supplementary Fig. A1). In many cases, the estimates for *σ*_*ε*_ and *ρ* had peaks close to 0. The 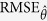 of the state estimates had either a bimodal distribution, or a long tail compared to that of the RMSE_*θ*_ (Supplementary Fig. A1). In other words, when the parameters were estimated, many replicates had much higher state estimate error than when the true parameter values were used (Fig. 1). In fact, 29.6% of the simulations had a 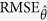 value that was 50% larger than their RMSE_*θ*_. When the simulations had high measurement error to process stochasticity ratios, the estimation problems for the states and two biologically relevant parameters, (*ρ*, *σ*_*η*_) were much higher (Fig. 1). The 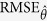 in some of these cases was close to 10 times greater than the simulated process stochasticity.

Our supplementary analyses demonstrated that similar estimation problems occurred when dlm and rjags were used (Supplementary information: Appendix C). However, while the parameters estimated with rjags were often biased, their distributions did not contain a peak at 0. Increasing the length of the time-series improved parameter and state estimation (Supplementary information: Appendix B). However, 500 time steps were insufficient to completely eliminate problems. Our supplementary analyses also show that the problems are less apparent when *ρ* is close to 1, or when we used the simpler non-stationary local-level model, which fixes the value of *ρ* = 1 (Supplementary information: Appendix A).

**Figure 2:**
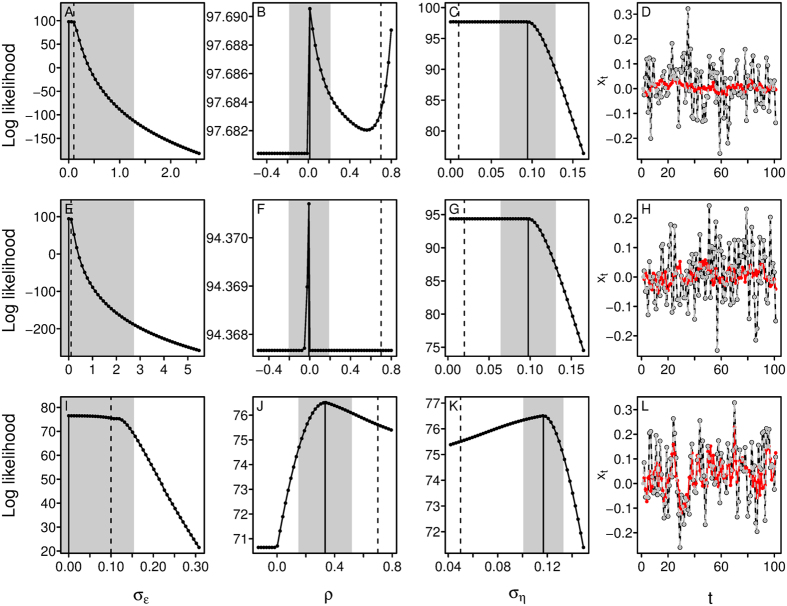
Log likelihood profiles for problematic simulations. In the first three columns, the curve represents the log likelihood when the focal parameter is fixed (the other parameters are optimise to maximise the log likelihood). The dash lines are the true parameter values (i.e., value used for the simulation), the full lines are the maximum likelihood estimates and the grey bands represent the 95% CI. The last column shows the time-series. The black lines represent the observations, *y*_*t*_, the red lines the simulated true states, *x*_*t*_, and the grey dashed lines the estimated states, 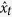.

The likelihood profiles of a subset of the problematic simulations revealed that the likelihood was flat in some areas and sometimes bimodal or jagged (Fig. 2). The CI of many parameters excluded the true simulated value. Because the estimated measurement error of these simulations were close to 0, the estimated states were very close to the observations and far from their true simulated values (Fig. 2D,H,L). When the states were fixed to their simulated rather than estimated values, the likelihood profiles were unimodal and most CI included the true parameter values, indicating that the problem lies in simultaneously estimating the states and the parameters (Supplementary information: Appendix D).

### Fixing the measurement error

Fixing the standard deviation of the measurement error to the simulated value, 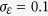, helped reduce the estimation problems (Supplementary information: Appendix G). 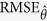 values were much closer to RMSE_*θ*_ when the measurement error was fixed rather than estimated. In this case, only 5.0% of the simulations had a 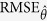 value that was 50% larger than their RMSE_*θ*_. However, fixing the measurement error did not completely resolve the estimation problems. Some parameter estimates continued to be on the boundary of parameter space and far from their simulated values. In addition, some likelihood profiles remained flat and some CIs spanned the entire parameter space (see Supplementary information: Appendix G for more detail).

### Ecological example

**Figure 3:**
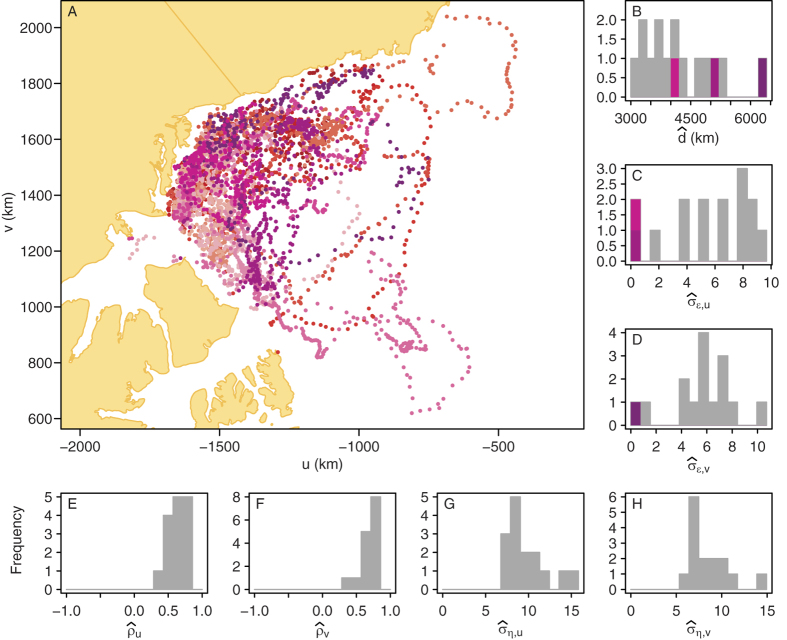
Polar bear movement, parameter estimates of the polar bear sea ice model, and estimates of the total voluntary bear displacement. (**A**) Locations of the 15 polar bears used in the analysis, with colours representing different individuals. The map was created in R^24^ using the Northern Hemisphere azimuthal equal-area EASE-Grid projection developed for polar sea ice data^41^. (**B**) Estimated total voluntary displacement over 342 days. (**C**–**H**) Parameter estimates of the polar bear sea ice models. The different colours in panels (**B**,**C**) represent the three individuals for which either 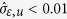 or 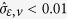.

The 15 polar bears studied used overlapping areas in the Beaufort Sea (Fig. 3A), but their parameters estimates varied widely (Fig. 3C–H). In particular, three individuals had much lower estimated sea ice measurement error, with either 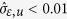 and 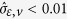. These three individuals had total voluntary displacement estimates that were on the higher end of the range (Fig. 3B). These results are similar to those found when we simulated movement data similar to the real polar bear data (Supplementary information: Appendix F). The simulations also showed that a few individuals would have 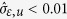 and 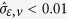 and that these individuals would be associated with higher values of total voluntary displacement.

## Discussion

Linear Gaussian SSMs, and approximations of them, are commonly used in the ecological literature to model animal movement^2^,^6^,^15^ and population abundance (e.g.^10^,^42^). These SSMs are often used to differentiate measurement error from process stochasticity and estimate the associated variance parameters (e.g.^10^,^13^,^42^,^43^). Our results demonstrated that simple linear Gaussian SSMs can have severe parameter- and state-estimation problems, and that these problems can affect biological inferences. According to our simulations, estimation problems were more frequent when the measurement error was much larger than the process stochasticity. In such cases, the three estimated parameters were often far from their simulated values, which in turn resulted in inaccurate state estimates. The ARMA notation shows that when the measurement error is much greater than the process stochasticity there is parameter redundancy, explaining why it is difficult to accurately estimate the parameters (Supplementary information: Appendix E). Our simulations showed that fixing the measurement error to its true value helped, but did not completely solve the estimation problems, especially when the fixed measurement error was relatively large. This is particularly worrisome because SSMs are most needed when the measurement error is large compared to the process stochasticity, and this is the condition under which the largest estimation problems occur.

The estimation problems are less critical when the measurement error is much smaller than the process stochasticity. While the measurement error estimates were often close to 0, the estimates for the other parameters, and those for the states, were generally accurate. As shown by the ARMA notation, when the measurement error is much smaller than the process stochasticity the model behaves as an AR(1) process, explaining why the measurement error estimates were often close to zero (Supplementary information: Appendix E). In effect, the measurement error is ignored. However, when the measurement error is negligible compared to the process stochasticity, ignoring the effect of the measurement error is less likely to affect our interpretation of the biological process.

Others have discussed estimation problems associated with fitting simple linear Gaussian SSMs. A few recent ecological studies have reported difficulties when estimating variance parameters, including variance estimates close to 0^17^,^44^. Dennis *et al.*^16^, who transformed the stochastic Gompertz population model into a linear Gaussian SSM, noted that while the process stochasticity and measurement error parameters can be estimated, multimodal likelihood functions occur and can lead to erroneous estimates. They showed that the likelihood functions tended to have multiple peaks, including two peaks associated with either no process stochasticity or no measurement error. While these two peaks can be local maxima, Dennis *et al.*^16^ noted that when there is substantial measurement error, one of these modes was often the global maximum. Knape^19^ extended the study of the Gompertz SSM to focus on the estimability of the density dependence parameter, an autocorrelation parameter similar to *ρ*. He found that the density dependence was generally not identifiable in the presence of unknown process variability and measurement error, especially when the strength of the density dependence was close to 0. When the measurement error was known, the strength of density dependence was estimable but the estimates often remained biased.

By extending the range of measurement error to process stochasticity ratios beyond those explored by Dennis *et al.*^16^ and Knape^19^, we demonstrate that relatively high measurement error can have dramatic effects on process parameter and state estimates, even when the measurement error is known. The results of Knape^19^ suggested that *ρ* values close to 0 would result in estimability problems (see also Forester *et al.*^45^), which is not surprising. As the process becomes less autocorrelated it is harder to differentiate it from the temporally independent measurement error, suggesting that differentiating between measurement error and process stochasticity would require a large sample size when *ρ* is far from 1. However, our results demonstrated that estimation problems remained with relatively high autocorrelation, *ρ* = (0.7, 0.99) and *ρ* fixed to 1, and relatively long time-series, *n* = (100, 500) (see Supplementary information: Appendices A-B). These results emphasize that the parameters and states are only estimable for a narrow range of conditions. Both the analysis of the ARMA formulation of our SSM and our ecological example show that parameter estimability within linear Gaussian SSMs is a general issue, not one restricted to the stochastic Gompertz population model. In fact, these problems extend to some nonlinear SSMs. For example, some of the estimated parameters of the nonlinear population SSMs of de Valpine and Hastings^46^ had considerable bias when measurement error was large relative to process variability, de Valpine and Hilborn^47^ showed that their advance Monte Carlo kernel likelihood method could not differentiate between the process stochasticity and measurement error of the nonlinear Schaefer population model, and Polansky *et al.*^20^ found similar problems in the theta-Ricker model.

Left undiagnosed, biased parameter estimates will mislead conclusions based on the problematic model parameters and may affect our interpretation of the other model parameters, the state estimates, and other derived values^11^,^48^. For example, stochastic population SSMs with negatively biased estimates of the process stochasticity will underestimate extinction risk^49^. In our polar bear example, erroneous estimates of measurement error and process stochasticity biased the state estimates and proxy for energy expenditure. Thus, even if the parameter values *per se* are not of interest, estimation problems need to be diagnosed because their effect on state estimates are likely to affect results of ecological importance.

The first step to avoid these biased inferences is to detect the potential for parameter estimability problems, which can be done through a variety of practical means. Our simulations demonstrated that estimates at the boundary of parameter space can be indicative of a problem. For our polar bear example, we detected the estimation problem because we had no reason to believe that the three bears with sea ice measurement error close to 0 used different sea ice than the other bears. These three bears were exposed to similar levels of sea ice drift as other bears and were not geographically or temporally isolated from them. Investigating the likelihood profile can also help detect estimation problems^16^,^50^,^51^. Indeed, the likelihood profiles of our problematic simulations had flat sections and multiple modes. However, in a Bayesian framework, the estimation problems can be obscured by the use of vague priors, as these can smooth the likelihood and affect inference^16^,^48^,^49^,^52^. When we used JAGS to estimates parameters, we had no estimates at the boundary and the posterior distributions of most parameters were unimodal, and yet, the estimates were biased (Supplementary information: Appendix C). A useful way to evaluate the model’s capacity to separate process and measurement error parameters, is to assess the extent of correlation between these estimates (see Supplementary information: Appendix H for details). In the maximum likelihood context, a plot of the likelihood surface can reveal a correlation pattern symptomatic of an identifiability issue^20^,^47^. In a Bayesian context, a plot of the joint posterior samples of these two parameters can reveal similar correlation patterns (Supplementary information: Appendix H). While few methods have been developed to formally assess parameter identifiability problems, data cloning^53^,^54^ and the symbolic method^4^,^55^ are promising avenues.

How can we avoid these estimability problems? In many cases, a larger sample size can help (see Supplementary information: Appendix B). In particular, Dennis *et al.*^50^ demonstrated that sampling replicates can substantially improve the capacity of SSMs to differentiate process stochasticity from measurement error, and that it may be advantageous to design monitoring programs with multiple replicate counts per survey rather than increasing the length of the time series (i.e., number of times the survey is conducted). However, for many observational studies, ecologists are limited in their ability to gather more data and, for movement data, it is often impossible to have replicates of location estimates. An alternative is to incorporate information on the measurement error. As we demonstrated in our simulation study, when we fix the measurement error to its true value, the estimates of the other parameters improved. While some parameter-estimation problems persisted, their effect on the state estimates diminished substantially. Similarly, de Valpine and Hilborn^47^ demonstrated that knowing the ratio of process to measurement variance would improve parameter estimates. In a Bayesian framework, specifying informative priors for the measurement error could help make the other parameters identifiable and improve the state estimates^11^,^49^ (but see Lele and Dennis^52^). Another alternative is to estimate the measurement error and process stochasticity outside of the SSM framework using the principle that the measurement error is uncorrelated over time whereas the process stochasticity is temporally correlated^56^. Estimating the measurement and process standard deviations offline reduces the number of parameters to estimate within the SSM framework. Using restricted maximum-likelihood, which treats fixed-effects parameters (e.g., *ρ*) and variance components (e.g., 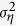, 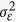) differently, can also be valuable to remove bias in SSM estimates^50^. When the estimation problem results in variance estimate close to 0, one can limit the estimate to interior (non-zero) solutions^16^,^19^. In particular, Dennis *et al.*^16^ suggested trying a variety of starting values for the optimizer used to numerically maximize the likelihood and eliminating all solutions that involve variance with near 0 values, even if one of these is the global maximum. Finally, restructuring the model can help reduce the problem. For example, in the polar bear example, we could create a population model with a single measurement error parameter for all bears. Even if the process variability continues to differ between individuals, using one measurement error term for all bears significantly decreases the number of parameters to estimate and increases the amount of data with which the measurement error term is estimated. As a general rule decreasing the number of parameters to estimate and increasing the amount of data will help reduce estimability problems.

Not all parameters are equally affected by estimation problems. Forester *et al.*^45^, who developed a linear Gaussian SSM for animal movement, demonstrated that coefficient parameters associated with covariates and an intercept in the measurement equation are easier to separate than process autocorrelation (equivalent to *ρ*), measurement error and process stochasticity. Note, however, that all of these parameters had cases associated with estimation problems. For example, the coefficient estimates were biased when their true simulated value was not equal to zero. Humbert *et al.*^57^ suggested that in the case of exponential growth SSMs the population trend parameter, similar to an intercept in the process equation, was often well estimated and that increasing the precision of the abundance estimates and the length of the time series, more than the completeness of the time series, could increase the performance of the SSM. This further indicates that ecologists should closely consider model formulation, and that the estimability of parameter should be assessed.

If we cannot resolve the parameter estimation problem, we need to account for its potential effect on our inference. One way to account for the estimation uncertainty is to use a parametric bootstrap to get CIs on the parameter and state estimates^16^,^45^. These bootstrap CIs require simulating the model using the estimated parameter values and re-fitting the model to each simulation. The 2.5^th^ and 97.5^th^ quantiles of the estimated parameters and states then becomes the 95% CI. These CIs differ from those we calculated from the standard deviation reported by TMB. However, because TMB is orders of magnitude faster than MCMC methods^13^, implementing these parametric bootstrap CIs would be computationally feasible, even for complex models. Note, however, that the variability in the estimates of our simulations suggests that these CIs would be large and would often approach the boundary of parameter space.

## Conclusion

We demonstrated that even simple linear Gaussian SSMs can have parameter estimability problems and that these problems can affect our ecological interpretation. As parameter estimability problems have been observed in other hierarchical models and because the ratio of information content to model complexity is expected to decrease with increasing numbers of hierarchies^48^,^52^, it is likely that these problems could occur in more complex forms of SSMs. Estimating individual variance components is notoriously difficult. SSMs do not escape this difficulty. While estimability problems have been discussed in the context of a few specific population dynamics SSMs (e.g.^16^,^19^,^20^), the voluminous literature on SSMs has paid relatively little attention to these problems. Such limited appreciation of the estimation problem is particularly dangerous because SSMs are usually advertised as providing the means to differentiate process from measurement variability (e.g.^2^,^46^,^58^).

It is timely to warn ecologists of these difficulties. SSMs are becoming the favoured framework for animal movement and population dynamics. SSMs used in ecology are becoming increasingly complex (e.g.^3^). In addition, tools to apply SSMs to data are becoming increasingly available. For example, R now provides a variety of packages that fit SSMs^59^. Until recently, SSMs were applied by statisticians or by ecologists with a strong statistical background. These researchers were more likely to be aware of potential estimability problems than most ecologists. Researchers have questioned whether ecologists have sufficient statistical training to properly implement hierarchical models and have suggested that universities should start including advanced courses in statistical modelling in their ecological programs (e.g.^16^,^60^). If the limitation of SSMs are not emphasized, the better accessibility of tools to fit these increasingly complex models are likely to lead to many undiagnosed estimation problems and incorrect conclusions.

While SSMs are powerful tools, they can give misleading results if they are misused. We believe it is important for ecologists to be aware of the potential estimation problems of SSMs. Investigating the likelihood profile, incorporating information on measurement error, and accounting for estimability uncertainty are all good first steps. However, we urge statisticians to develop further tools that can be used to diagnosed such problems and these should be readily available along with the tools to fit SSMs.

## Additional Information

**How to cite this article**: Auger-Méthé, M. *et al.* State-space models' dirty little secrets: even simple linear Gaussian models can have estimation problems. *Sci. Rep.* **6**, 26677; doi: 10.1038/srep26677 (2016).

## Supplementary Material

Supplementary Information

Supplementary Dataset
