# Supplementary material for: State-space models’ dirty little secrets: even simple linear Gaussian models can have estimation problems
Source: Sci Rep. 2016 May 25;6:26677. doi: 10.1038/srep26677 (PMC4879567; doi:10.1038/srep26677)
Supplement: Supplementary Information [file srep26677-s1.pdf]

Supplementary Information - State-space models' dirty little secrets: even simple linear Gaussian models can have estimation problems

Marie Auger-Méthé[1,*], Chris Field[1], Christoffer M. Albertsen[2], Andrew E. Derocher[3], Mark A. Lewis[3,4], Ian D. Jonsen[5], and Joanna Mills Flemming[1]

[1]Dalhousie University, Department of Mathematics and Statistics, Halifax
[2]Technical University of Denmark, National Institute of Aquatic Resources
[3]University of Alberta, Department of Biological Sciences
[4]University of Alberta, Department of Mathematical and Statistical Sciences
[5]Macquarie University, Department of Biological Sciences
[*]auger-methe@dal.ca

A Some problems persist when ρ is close to 1 and in the local-level model

Knape,[1] who investigated a linear Gaussian SSM describing the stochastic Gompertz population model, found that an autocorrelation parameter similar to ρ was harder to estimate when its true simulated value was close to 0. ρ appears to be problematic especially when $|\rho| < 0.5$. Similarly, Forester et al.,[2] who developed a linear Gaussian SSM for animal movement, noticed that when the autocorrelation was close to 1 (i.e., 0.95) there was less estimation problems than when it was close to 0 (i.e., 0 or 0.2). This is not surprising. As the process becomes less autocorrelated it is harder to differentiate it from the temporally independent measurement error. As such, we focussed on investigating whether the estimation problems remained when the autocorrelation parameter was relatively high. In the main text, we have presented the results when $\rho = 0.7$. In this Appendix, we investigated higher ρ values. First, we recreated the same simulation study as in section Demonstration of the problem of the main text, except that we used $\rho = 0.99$ in the simulations. Second, we used a simpler model called the local model, which is sometimes referred as the random walk plus noise (e.g.,[3]):

$$\text{Measurement eq} \qquad y_t = x_t + \varepsilon_t\,, \qquad \varepsilon_t \sim N(0, \sigma_\varepsilon^2)\,, \text{ where } t \geq 1, \sigma_\varepsilon^2 > 0 \tag{1}$$

$$\text{Process eq} \qquad x_t = x_{t-1} + \eta_t\,, \qquad \eta_t \sim N(0, \sigma_\eta^2)\,, \text{ where } t \geq 1, \sigma_\eta^2 > 0. \tag{2}$$

The only difference with the model presented in eq. 1-2 is that there is no ρ parameters, which is the equivalent of fixing $\rho = 1$. Note that while this simpler model has fewer parameters to estimate, it is no longer stationary.[4] Following the methods described in section Demonstration of the problem, we simulated and fitted this simpler model.

The parameter and state estimates improved in simulations with $\rho = 0.99$ (Fig. A.2). In particular, the estimates for the process parameters (ρ, σ_η) were much closer to their simulated values than when the simulated ρ value was 0.7 (e.g., compare

Fig. A.2B-C to Fig. A.1B-C). In some cases, this translated in better state estimates (e.g., compare Fig. A.2H to Fig. A.1H). Similarly, using the local model improved parameter and states estimates (Fig. A.3). However, this did not completely eliminate the estimation problems. When the measurement error was much larger than the process stochasticity, $\sigma_\varepsilon = 10\,\sigma_\eta$, some of the parameter estimates remained on the boundary of parameter space (e.g., Fig. A.2A and Fig. A.3B). In the case of simulations with $\rho = 0.99$, the estimated ρ was close to 0 (Fig. A.2B,F) and some state estimates were far from those estimated when the parameter values were known (e.g, Fig. A.2D,H).

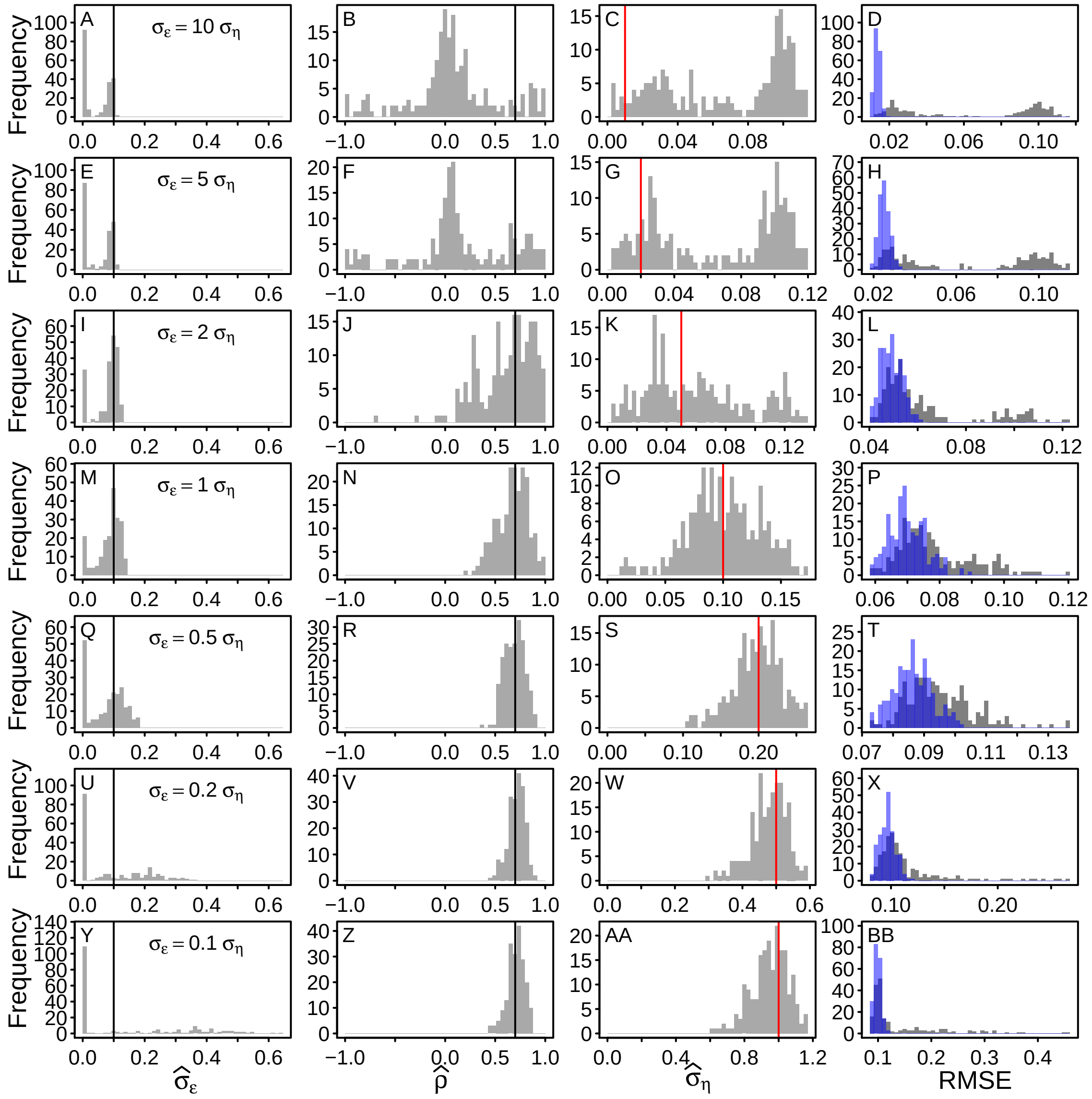

Figure A.1. Histograms of the parameter estimates and of the RMSE of the state estimates when $\rho = 0.7$. This is a more detailed visualization of the results presented in Fig. 1 of the main text. Each row represents the results of 200 simulations for a set of parameter values. For the first three columns, the vertical lines represent the parameter values used in the simulations, with black lines used for values that remained constant, $\sigma_\varepsilon = 0.1$ and $\rho = 0.7$, and red lines for values that changed between sets, $\sigma_\eta = (0.01, 0.02, 0.05, 0.1, 0.2, 0.5, 1)$. In the last column, the grey histograms represent the RMSE of the model fitted using the estimated parameter values, while the blue histograms represent the RMSE when the model is fitted using the true values.

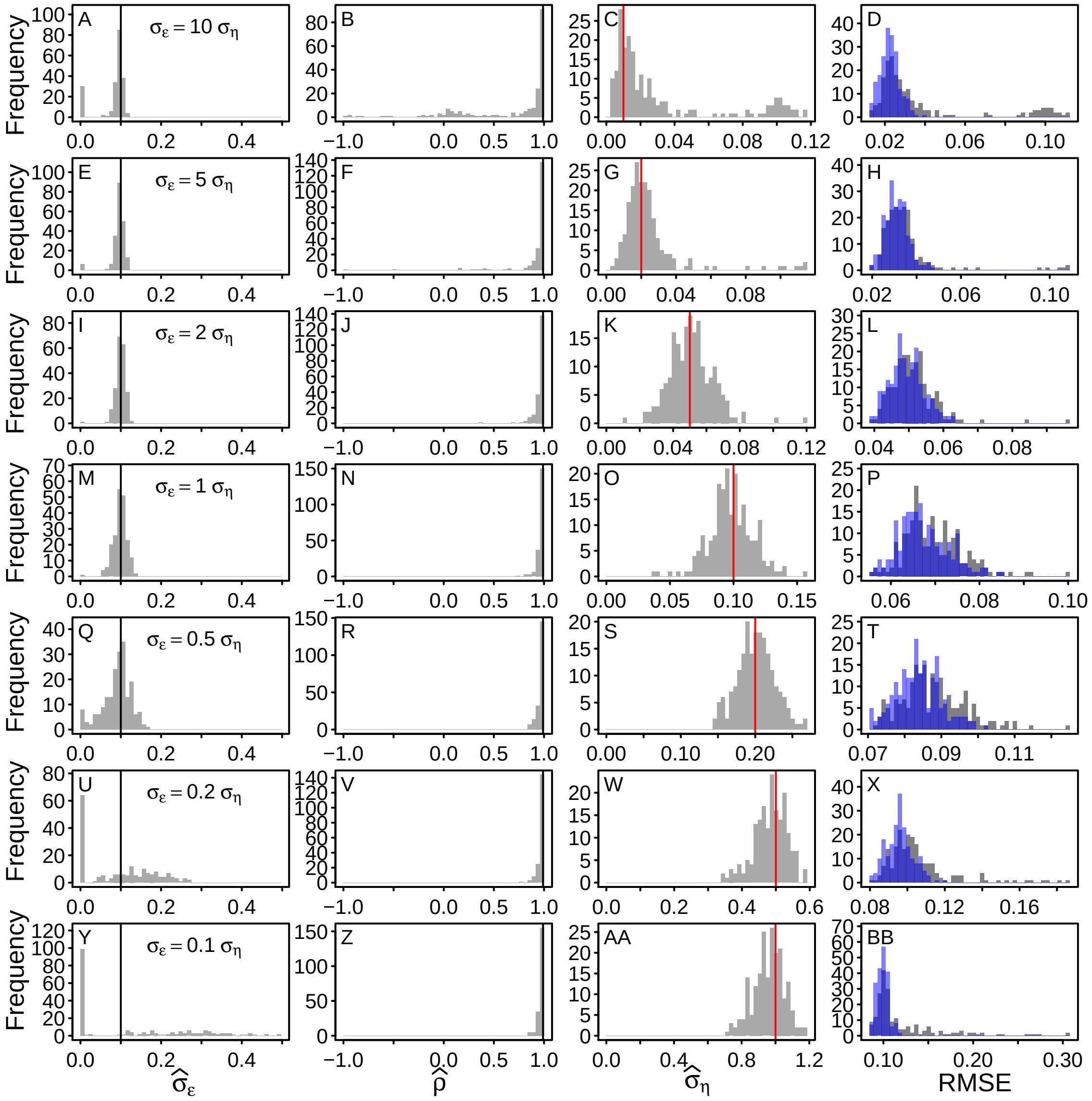

Figure A.2. Histograms of the parameter estimates and of the RMSE of the states when $\rho = 0.99$. Each row represents the results of 200 simulations for a set of parameter values. For the first three columns, the vertical lines represent the parameter values used in the simulations, with black lines used for the values that remain constant for all simulation sets, $\sigma_\varepsilon = 0.1$ and $\rho = 0.99$, and red lines for values that change between set, $\sigma_\eta = (0.01, 0.02, 0.05, 0.1, 0.2, 0.5, 1)$. In the last column, the grey histograms represent the RMSE of the model fitted using the estimated parameter values, while the blue histograms represent the RMSE when the model is fitted using the simulation values.

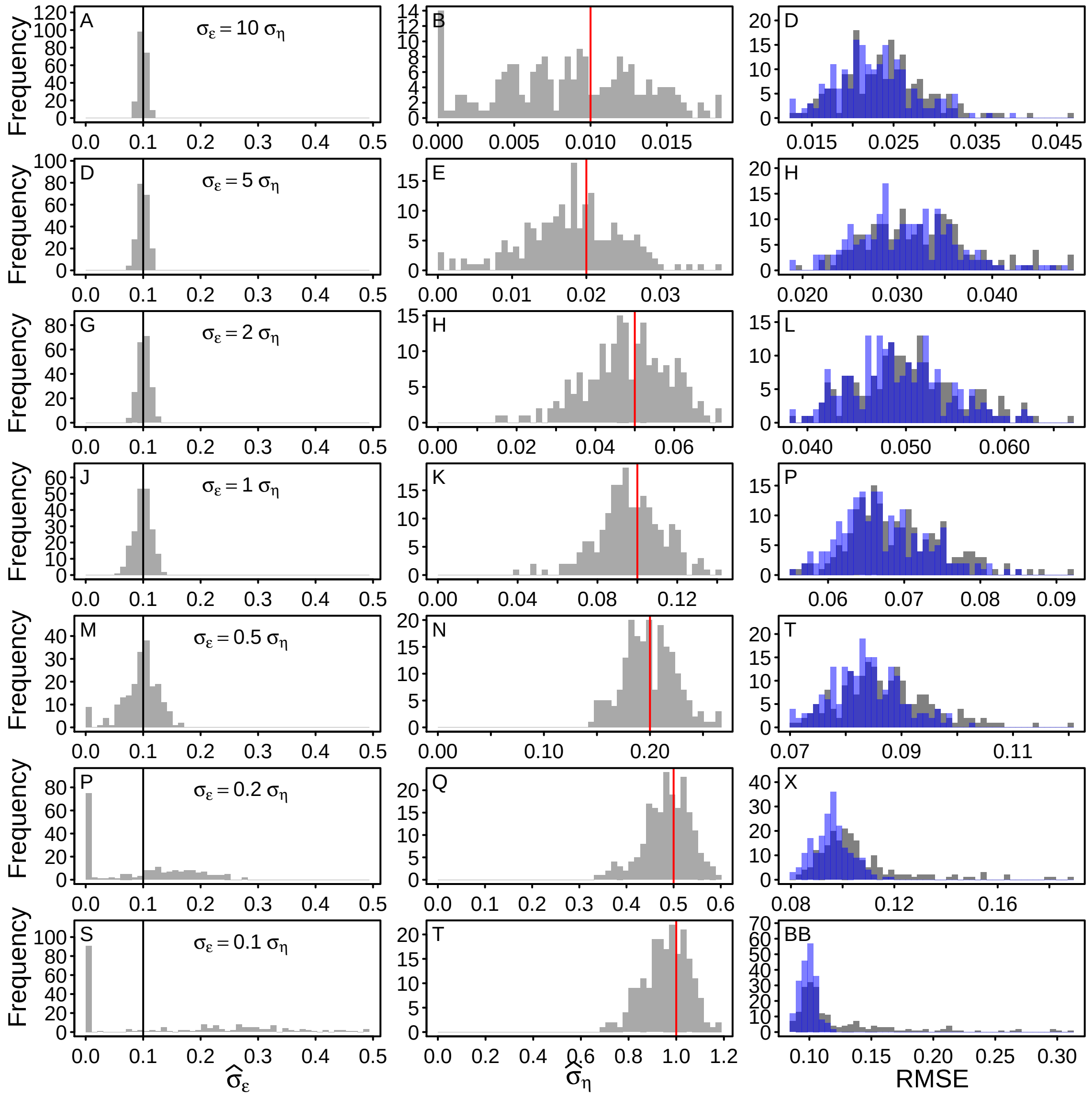

Figure A.3. Histograms of the parameter estimates and of the RMSE of the states for a set of simulations of the local-level model. Each row represents the results of 200 simulations for a set of parameter values. For the first three columns, the vertical lines represent the parameter values used in the simulations, with black lines used for the values that remain constant for all simulation sets, $\sigma_\varepsilon = 0.1$, and red lines for values that change between set, $\sigma_\eta = (0.01, 0.02, 0.05, 0.1, 0.2, 0.5, 1)$. In the last column the grey histograms represent the RMSE of the model fitted using the estimated parameter values, while the blue histograms represent the RMSE when the model is fitted using the simulation values.

B Some problems persist with longer time-series

Using longer time-series can considerably reduce estimability problems. In this appendix, we investigate whether the estimation problem persisted with longer time-series. We wanted to use a length of time-series that is relevant to real ecological examples, keeping in mind that, generally, population abundance time-series are shorter than movement time-series. For example, a recent population study using bird count data was limited to 45 to 52 counts.[5] As such previous simulation studies for population dynamics SSMs limited their time-series to 30 and 100 time steps.[1,6,7] In fact, Dennis et al.[6] mentioned that times-series of 100 time steps were unrealistic for ecological data of the type and thus our example in the main text ($n = 100$) is likely underestimating the frequency of estimation problems. Movement time-series are generally of longer length. For example, the simulation study of Forester et al.[2] used 350 steps and their real movement data ranged from 265-390 locations. Our polar bear time-series ranged from 342 to 365. In addition, with technological advancements movement time-series are becoming much longer. Thus, to look at time-series more representative of movement time-series we conducted the same simulation analysis as in section Demonstration of the problem of the main text, with the only difference being that our time-series have 500 observations ($n = 500$) rather than 100 ($n = 100$).

When time-series of 500 steps were used, the estimation problems were reduced (compare Fig. B.1 to Fig. A.1). In particular, when $\sigma_\varepsilon \leq 2\,\sigma_\eta$, we had fewer σ_ε estimates at the boundary of parameter space (e.g., compare Fig. B.1Q to Fig. A.1Q) and the estimates of σ_η and ρ are closer to the simulated values (e.g., compare Fig. B.1N,O to Fig. A.1N,O). However, when the measurement error is large compared to the process stochasticity, $\sigma_\varepsilon \geq 5\,\sigma_\eta$, many σ_ε estimate remained at the boundary of parameter space (Fig. B.1A,E), many σ_η estimates were positively biased (Fig. B.1C,G), and many ρ estimates were negatively biased, with values close to 0 (Fig. B.1B,F). In addition, when the parameters were estimated, many replicates had higher state estimate error than when the true parameter values were used (Fig. B.1D,H), indicating that biases in parameter estimates continued to affect the state estimates. Overall, these results suggest that longer time-series do improve the estimability of some parameters and states, but that to have reliable estimates when the measurement error is much larger than the process stochasticity would require much longer time-series.

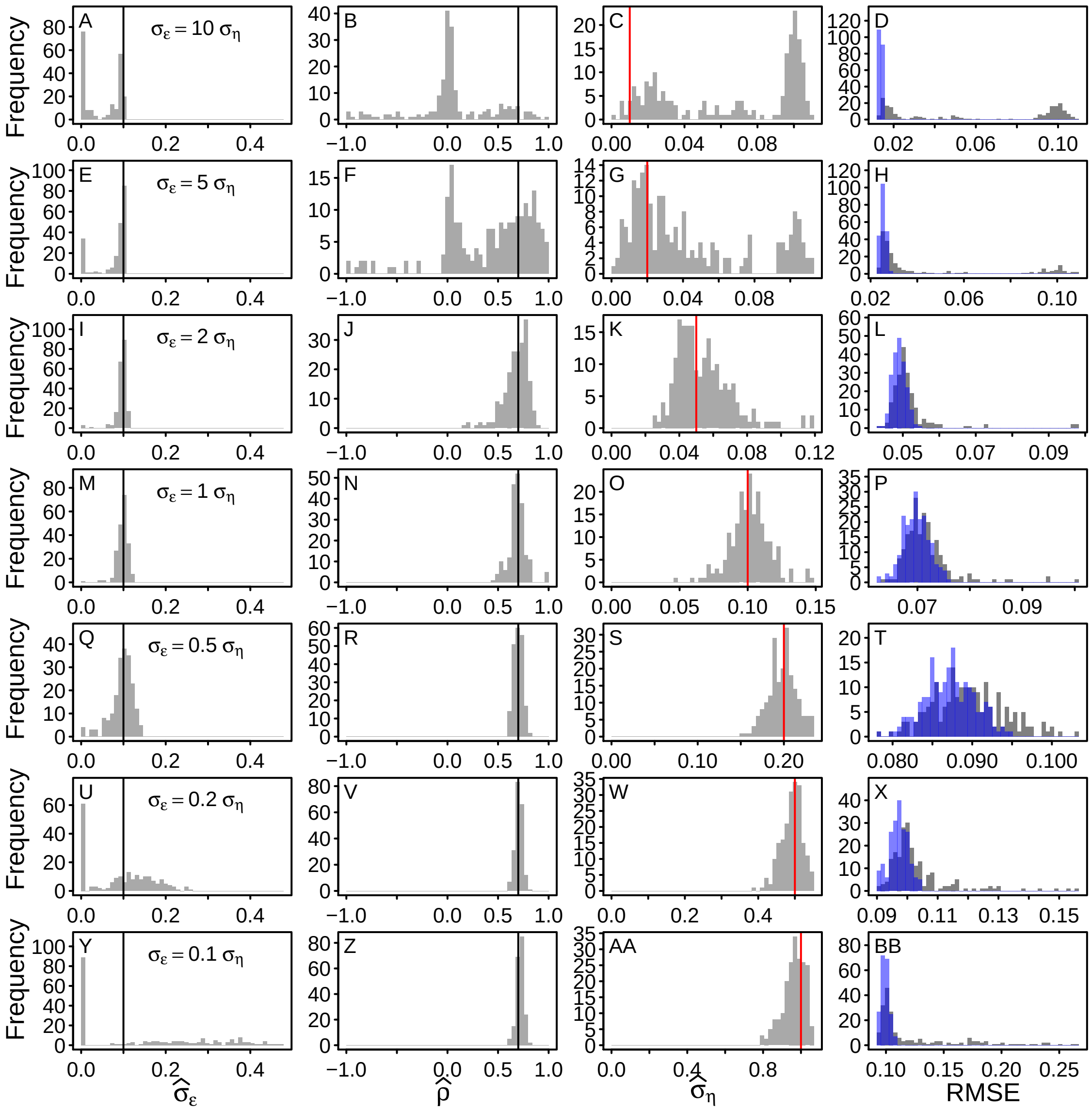

Figure B.1. Histograms of the parameter estimates and of the RMSE of the states for a set of simulations with 500 time steps. Each row represents the results of 200 simulations for a set of parameter values. For the first three columns, the vertical lines represent the parameter values used in the simulations, with black lines used for the values that remain constant, $\sigma_\varepsilon = 0.1$ and $\rho = 0.7$, and red lines for values that change between set, $\sigma_\eta = (0.01, 0.02, 0.05, 0.1, 0.2, 0.5, 1)$. In the last column, the grey histograms represent the RMSE of the model fitted using the estimated parameter values, while the blue histograms represent the RMSE when the model is fitted using the simulation values.

C Results with other R packages

We chose to use TMB for most analyses because it is a fast and flexible package that can be used to fit a variety SSMs to data (e.g.,[8]). To verify that the estimation problems are not limited to this package and are general problems associated with linear Gaussian SSMs, we also used two additional packages to reproduce the simulation study explained in section Demonstration of the problem of the main text. First, we used dlm,[3] a package that was used in recent ecological studies to fit SSMs to data.[5,9] The package dlm maximises the log likelihood numerically and has functions to estimate the states via Kalman filter and smoother. To be consistent with TMB, we used the Kalman smoother, which takes into account all observations (see[8]).

Second, we used rjags,[10] which is an R interface to JAGS, a program that allows for the analysis of Bayesian hierarchical models using Markov Chain Monte Carlo (MCMC) methods. Unlike TMB and dlm, rjags requires the specification of priors for the estimated parameter. We used the vague priors: $\sigma_\varepsilon \sim \text{HalfN}(0, \sigma^2 = 10000)$, $\rho \sim \text{Uniform}(0,1)$, and $\sigma_\eta \sim \text{HalfN}(0, \sigma^2 = 10000)$. We used two chains, each with 50 000 adaptation steps, 50 000 burn in steps and 50 000 saved steps. For each chain we kept 1 every 500 steps.

The results when we used dlm are nearly identical to those when we used TMB (compare Fig. C.1 to Fig. A.1). The only difference, is that when $\sigma_\varepsilon \geq 5\ \sigma_\eta$ a few less replicates had σ_ε values close to 0, ρ values close to 0, and positively biased σ_η values. However, these differences were small and some were accompanied by other biases, such as more ρ values close to -1. Overall, the conclusion made for TMB in the main text hold true for dlm.

In contrast, the results from rjags are different from TMB. In particular, the distribution of estimates were unimodal and there was no estimates close to the boundary of parameter space (i.e., no $\widehat{\sigma}_\varepsilon$ close to 0). However, the peak of estimates was often far from the simulated value (Fig. C.3), indicating that both parameter and state estimates were often biased. This is potentially due to the fact that vague priors can influence the results and smooth the peculiarities of the likelihood.[6] This effect could explain why estimation problems have been less easily detected in recent SSMs studies, which often uses complex Bayesian SSMs. In addition, the posterior distributions were more unimodal and not as flat as the likelihood profiles produced by likelihood-based methods (compare Fig. C.3 to Fig. 2). Note that these results exclude the 37 replicates out of 1400 simulations that did not converge (scale reduction factor of any parameter > 1.1), and thus would have been deemed problematic with such metrics. Overall, the results from JAGS indicate that using Bayesian methods does not fix the estimation problems of the linear Gaussian SSMs, and in fact might have made them harder to detect. See Appendix H for a more detailed discussion of diagnostic tools.

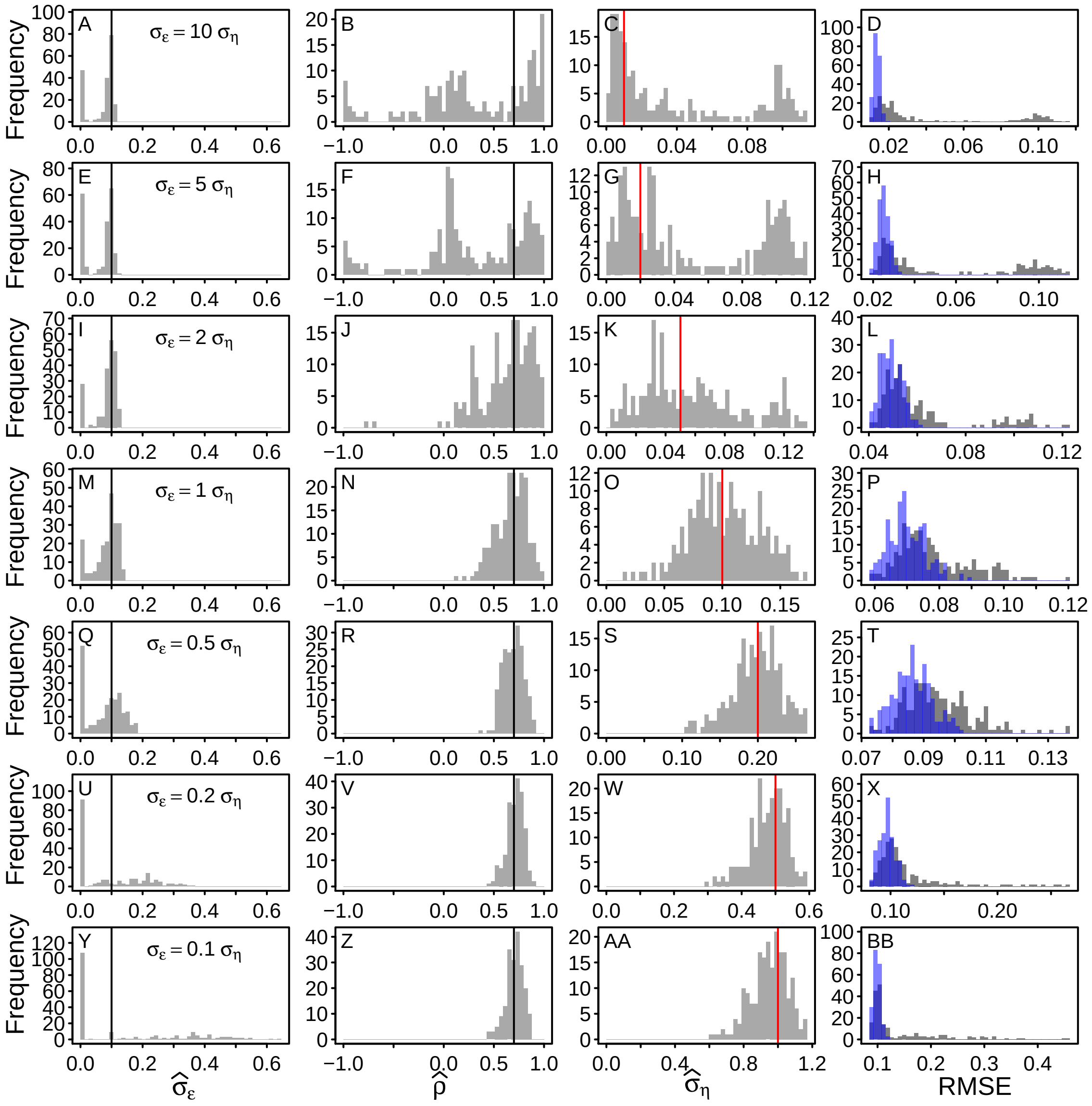

Figure C.1. Histograms of the parameter estimates and of the RMSE of the states when `dlm` is used to fit our SSM to a set of simulations. Each row represents the results of 200 simulations for a set of parameter values. For the first three columns, the vertical lines represent the parameter values used in the simulations, with the black lines used for the values that remain constant, $\sigma_\varepsilon = 0.1$ and $\rho = 0.7$, and the red lines for values that change between set, $\sigma_\eta = (0.01, 0.02, 0.05, 0.1, 0.2, 0.5, 1)$. In the last column, the grey histograms represent the RMSE of the model fitted using the estimated parameter values, while the blue histograms represent the RMSE when the model is fitted using the simulation values. Note that the parameters of 2 out of the 1400 simulations could not be estimated due to singularity problems.

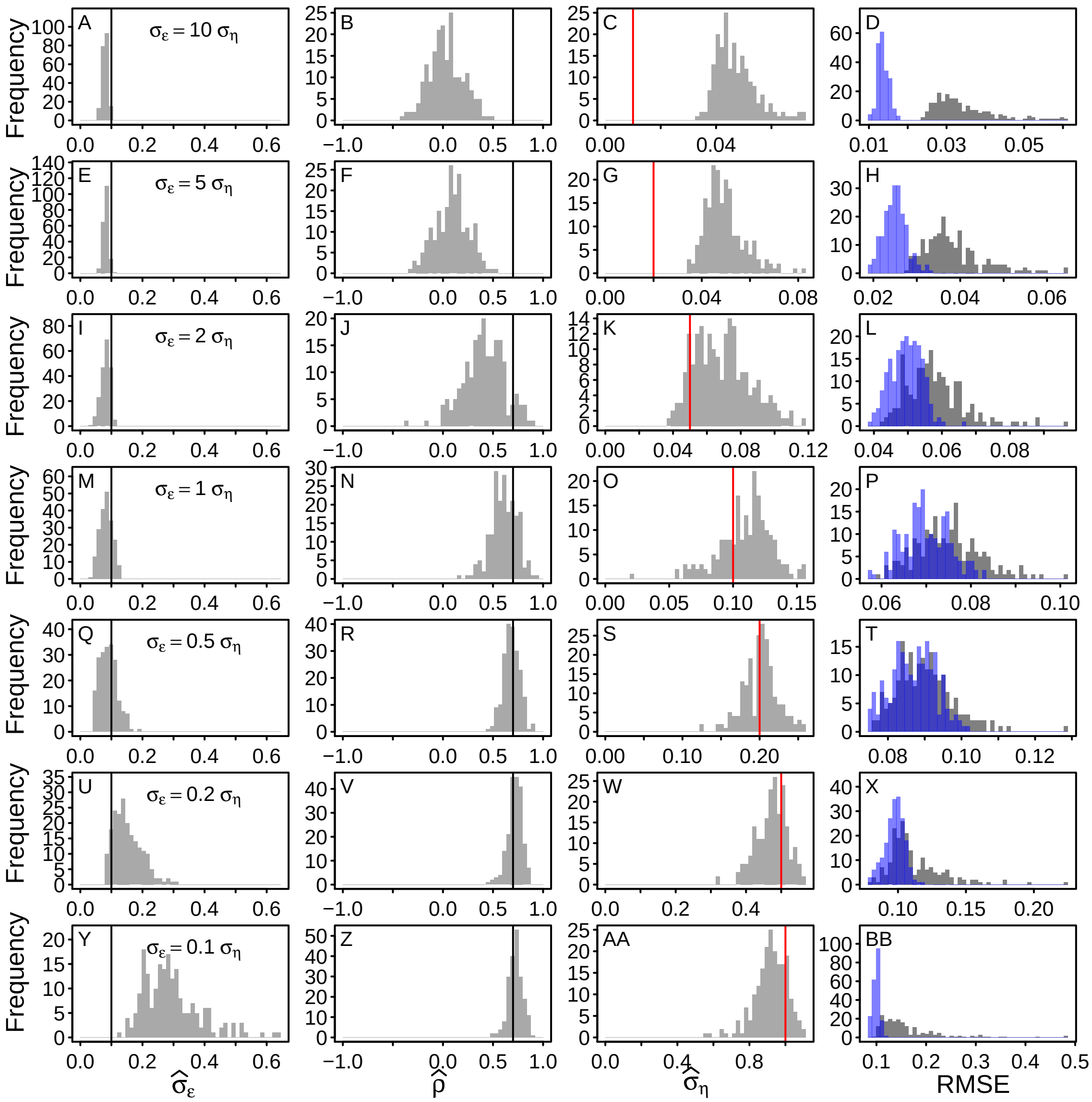

Figure C.2. Histograms of the parameter estimates and of the RMSE of the states when `rjags` is used to fit our SSM to a set of simulations. Each row represents the results of 200 simulations for a set of parameter values. For the first three columns, the vertical lines represent the parameter values used in the simulations. The black lines are used for the values that remain constant for all simulation sets: $\sigma_\varepsilon = 0.1$ and $\rho = 0.7$. The red line for that values that change in between set: $\sigma_\eta = (0.01, 0.02, 0.05, 0.1, 0.2, 0.5, 1)$. In the last column the grey histograms represent the RMSE of the model fitted using the estimated parameter values, while the blue histograms represent the RMSE when the model is fitted using the simulation values. Note that 37 out of 1400 simulations did not converged (i.e. the potential scale reduction factor was > 1.1 for one of the parameters).

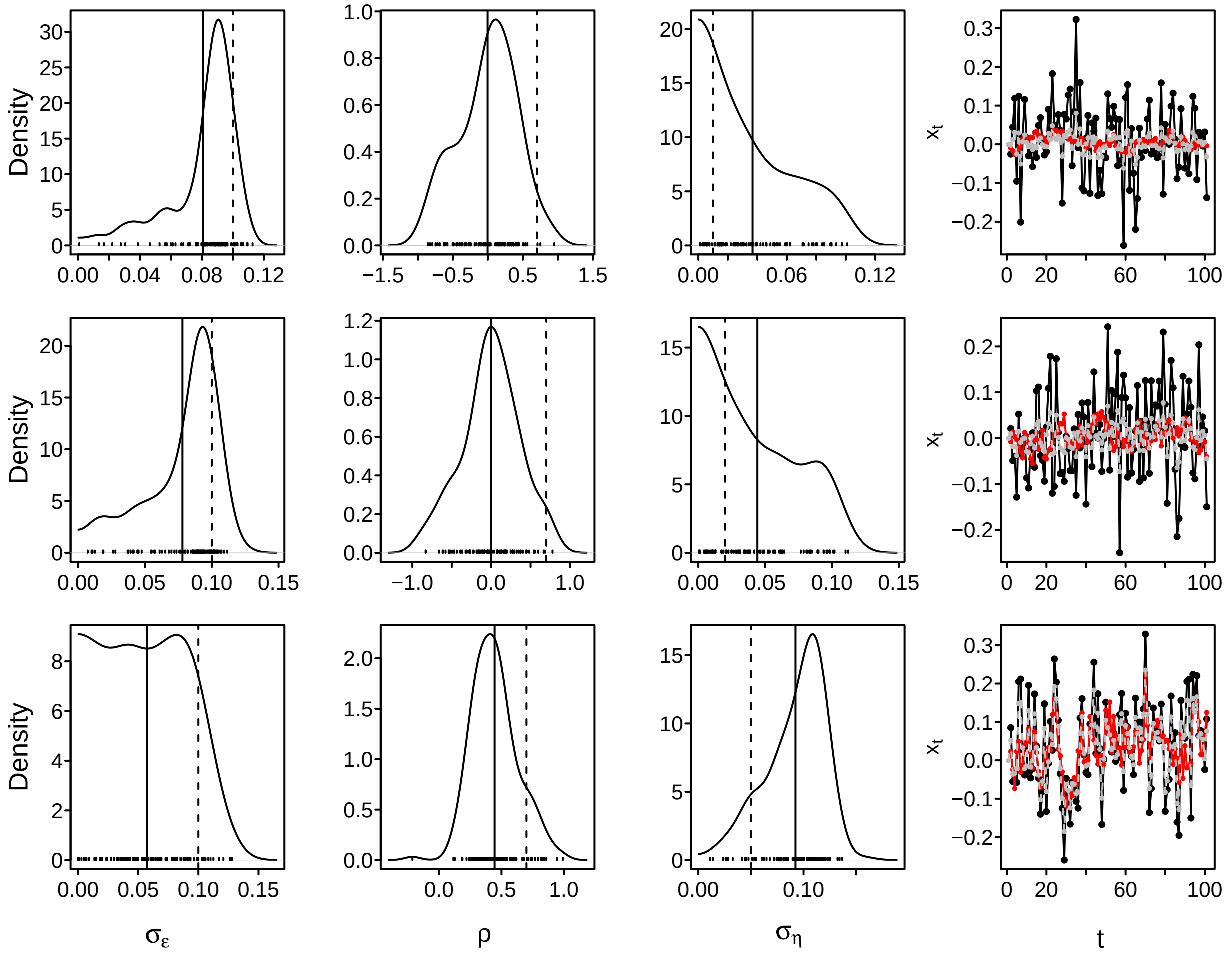

Figure C.3. Posterior distribution for problematic simulations. The first three columns, the curve represents the posterior distribution for the estimated parameters. The dash lines are the true parameter values (i.e., value used for the simulation), the full lines are the mean value. The last column shows the time-series. The black lines represent the observations, y_t, the red lines the simulated true states, x_t, and the grey dashed lines the estimated states, $\hat{x}_t$. Note that these three simulations converged (the potential scale reduction factor was < 1.1 for all parameters).

D Estimating parameters when we know the true states

In this Appendix we investigate whether the parameter-estimation problems are associated with estimating the parameters at the same time as the states. To do so, we estimated the parameters when the states were fixed to their true simulated values. We focused on the same problematic simulations as those explored in the main text. When the states were known, the parameter estimates were close to the simulated values and the CIs included the true simulated values (Fig. D.1). In addition, the likelihood profiles were unimodal, demonstrating the type of likelihood profiles one would expect for well-behaved models. These results suggest that the problems lie in estimating both the states and the parameters.

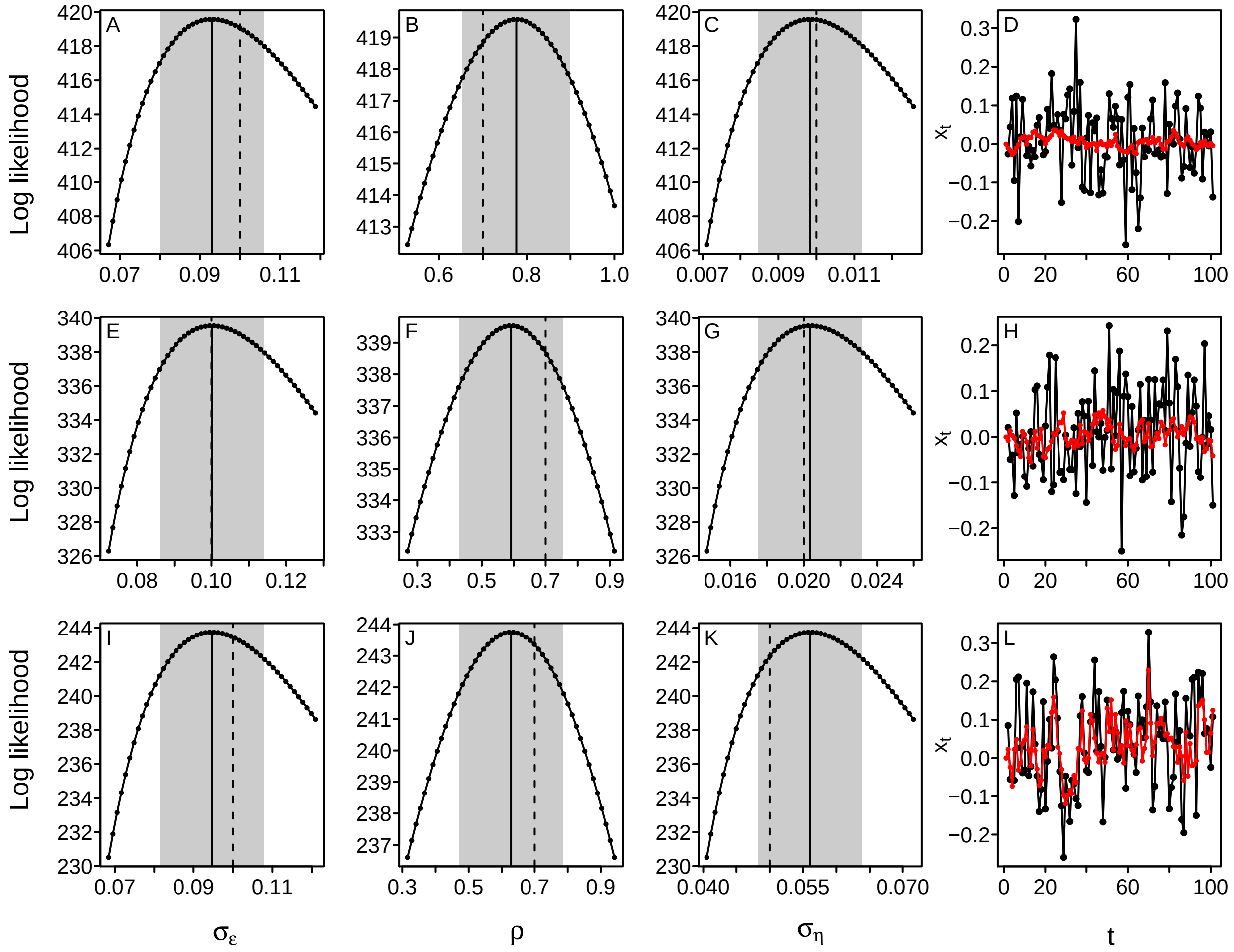

Figure D.1. Log likelihood profile for problematic simulations when the state values are fixed to the simulated values. In the first three columns, the curve represents the log likelihood when the focal parameter is fixed (the other parameter are optimize to maximize the log likelihood). The dash lines are the true parameter values (i.e., value used for the simulation), the full lines are the maximum likelihood estimates and the grey bands represent the 95% CIs. The last column shows the time-series. The black lines represent the observations, y_t, and the red lines the simulated true states, x_t. Note that these are the same simulations as in Fig. 2.

E Reformulating our simple SSMs with the ARMA notation

For certain parameter values, the SSM can behave either as a white noise process or as an AR(1) process. In both cases, the SSM formulation of the model will be over-parameterized, and will lead to estimation problems. To see this, we can rewrite our SSM as an ARMA(1,1) model. First, we can combine eq. 1 and 2 and reparametrize the model in terms of ε_t:

$$y_t = \rho x_{t-1} + \eta_t + \varepsilon_t, \qquad \varepsilon_t \sim N(0, \sigma_\varepsilon^2), \eta_t \sim N(0, \sigma_\eta^2) \tag{3}$$

$$= \rho(x_{t-1} + y_{t-1} - y_{t-1}) + \eta_t + \varepsilon_t \tag{4}$$

$$= \rho y_{t-1} + \rho \varepsilon_{t-1} + \eta_t + \varepsilon_t \tag{5}$$

Since η_t and ε_t are independent and normally distributed, their sum, $v_t = \eta_t + \varepsilon_t$, follows a normal distribution with zero mean and variance $\sigma_v^2 = \sigma_\eta^2 + \sigma_\varepsilon^2$. Now, if we let $v_{t-1} \sim N(0, \sigma_v^2)$, we can rescale its variance such that:

$$y_t \stackrel{D}{=} \rho y_{t-1} + \rho \frac{\sigma_\varepsilon}{\sqrt{\sigma_\varepsilon^2 + \sigma_\eta^2}} v_{t-1} + v_t. \tag{6}$$

This is an ARMA(1,1) process with AR parameter $\phi = \rho$, MA parameter $\psi = \rho \frac{\sigma_\varepsilon}{\sqrt{\sigma_\varepsilon^2 + \sigma_\eta^2}}$, and variance parameter σ_v^2.

When $\sigma_\varepsilon \ll \sigma_\eta$, then ψ is small. Thus the process behaves as an AR(1) process with parameters ρ and σ_η. This is the case for our simulations with $\sigma_\eta = 0.5, 1$ (Fig. A.1). When $\sigma_\varepsilon \gg \sigma_\eta$, then $\psi \approx \phi$ and hence there is parameter redundancy in the model.[11] In this case, the process closely resembles white noise. This is the case for our most problematic simulations ($\sigma_\eta = 0.01, 0.02, 0.05$, see Figs. A.1-2).

F Polar bear and sea ice simulations

To demonstrate that the polar bear and sea ice model has estimation problems and show how these problems may affect our interpretation of our proxy of energy expenditure, we simulated movement using model described in eq. 6-8. We simulated 500 movement paths with $n = 342$ using the parameters estimated with the polar bear data (Table F.1). We also used the initial state values estimated with the polar bear data: $\mathbf{x}_0 = \begin{bmatrix} 2.01 \\ -4.08 \end{bmatrix}$. We simulated the sea ice displacement in the u- and v-direction using normal distributions with mean and standard deviation values based on the sea ice drift experienced by the polar bears in our sample: $s_{t,u} \sim N(\mu = 1.49, \sigma = 4.97)$ and $s_{t,v} \sim N(\mu = 2.26, \sigma = 3.97)$. As for the empirical data, we estimated parameters and the total voluntary displacement (see eq. 10).

Table F.1. Parameter estimates for the polar bear sea ice empirical data. These are the parameter values used in the simulations.

Parameters	mean	sd
$\sigma_{\varepsilon,u}$	5.53	3.10
$\sigma_{\varepsilon,v}$	5.79	2.66
ρ_u	0.635	0.128
ρ_v	0.685	0.123
$\sigma_{\eta,u}$	9.66	2.47
$\sigma_{\eta,v}$	8.56	2.33

Our simulation results show that the model appears to have similar estimation problems than the simple SSM extensively studied in the manuscript. In particular, we have a few simulations where the estimates of $\sigma_{\varepsilon,u}$ and $\sigma_{\varepsilon,v}$ are close to 0 (Fig. F.1), something that was also noticeable in the empirical data (Fig. 3). Parameter estimates close to 0 can be associated with estimation problems, which in turn can affect the state estimate and thus the estimates of the total displacement (Fig. F.2). In particular, simulations with either $\widehat{\sigma}_{\varepsilon,u} < 0.01$ or $\widehat{\sigma}_{\varepsilon,v} < 0.01$ tended to be associated with higher d values (Fig. F.2). The estimates of d ranged widely in values, but appeared to by generally higher.

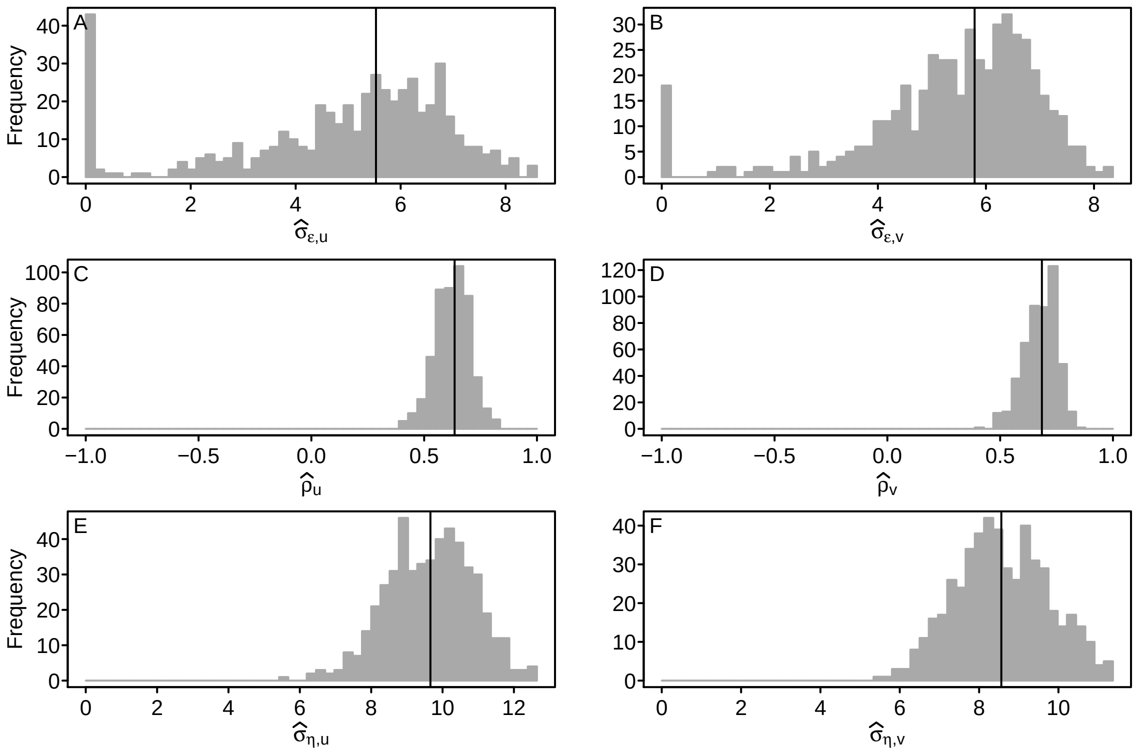

Figure F.1. Histograms of the parameter estimates for the set of 500 simulations of the polar bear sea ice model. The vertical lines represent the parameter values used in the simulations. The black lines represent the value used to simulate the data (see Table F.1).

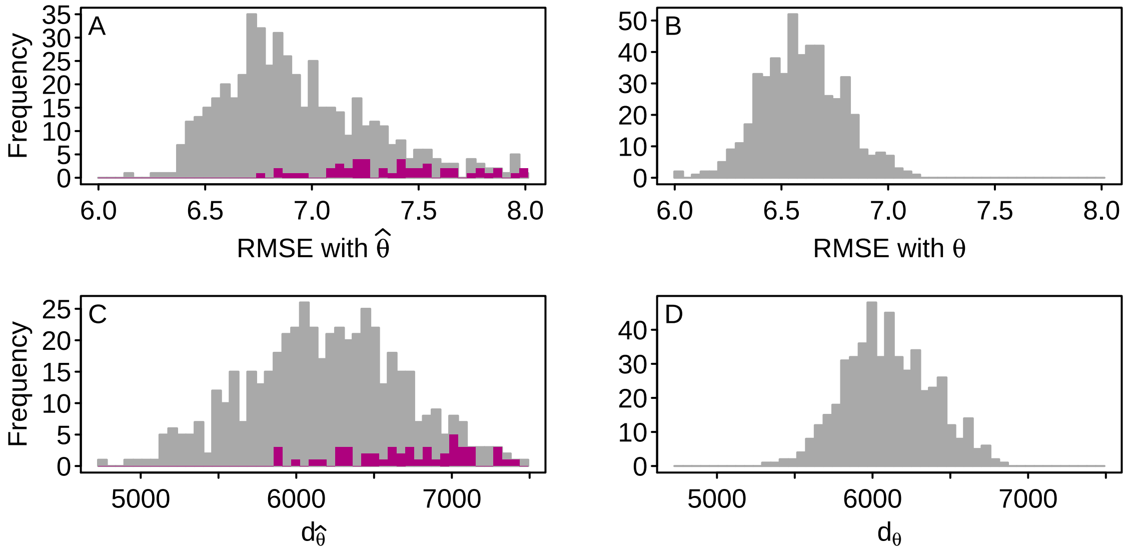

Figure F.2. Histograms of the RMSE of the states for the polar bear sea ice model and of the total displacement, d. The left column represents results when the model was fitted using the estimated parameter values. The right column represents the results when the model was fitted using the simulation values. The purple columns represent the simulations for which either $\widehat{\sigma}_{\varepsilon,u} < 0.01$ or $\widehat{\sigma}_{\varepsilon,v} < 0.01$.

G Results when the measurement error is fixed

As explained in section Incorporating measurement error information of the main text, we investigated whether fixing the measurement error resolved the parameter estimation problem. To do so, we fitted our simple likelihood (equation 4) to the same simulations as in section Demonstration of the problem, but we fixed the standard deviation of the measurement equation to the value used to simulate the data, $\sigma_\varepsilon = 0.1$. We only estimated the remaining parameters, $\theta_{\mathbf{m}} = (\rho, \sigma_\eta)$. As for the main analysis, we investigated the parameter estimates, RMSE of the states, and likelihood profiles. In addition, we explored the likelihood surfaces.

As mentionned in section Fixing the measurement error of the main text, fixing the standard deviation of the measurement error to the simulated value, $\sigma_\varepsilon = 0.1$, helped reduce the estimation problems (compare Fig. G.1 to Fig. A.1). In particular, the state RMSE when the parameters were estimated were much closer to those when the parameters were fixed to the simulated values (e.g., compare Fig. G.1D to Fig. A.1D). In this case, only 5.0% of the simulations had a RMSE$_{\hat{\theta}}$ value that was 50% larger than their RMSE$_\theta$. In addition, likelihood profiles were more unimodal than when all parameters were estimated (e.g., compare Fig. G.2J to Fig. 2J), and the state estimates were no longer simply echoing the observations (e.g., compare Fig. G.2L to Fig. 2L). However, using measurement error information did not completely resolve the estimation problems. Some parameter estimates continued to be on the boundary of parameter space and far from their simulated values (e.g., Fig. G.1E). In addition, some likelihood profiles remained flat and some CIs spanned the entire parameter space (e.g., Fig. G.2B).

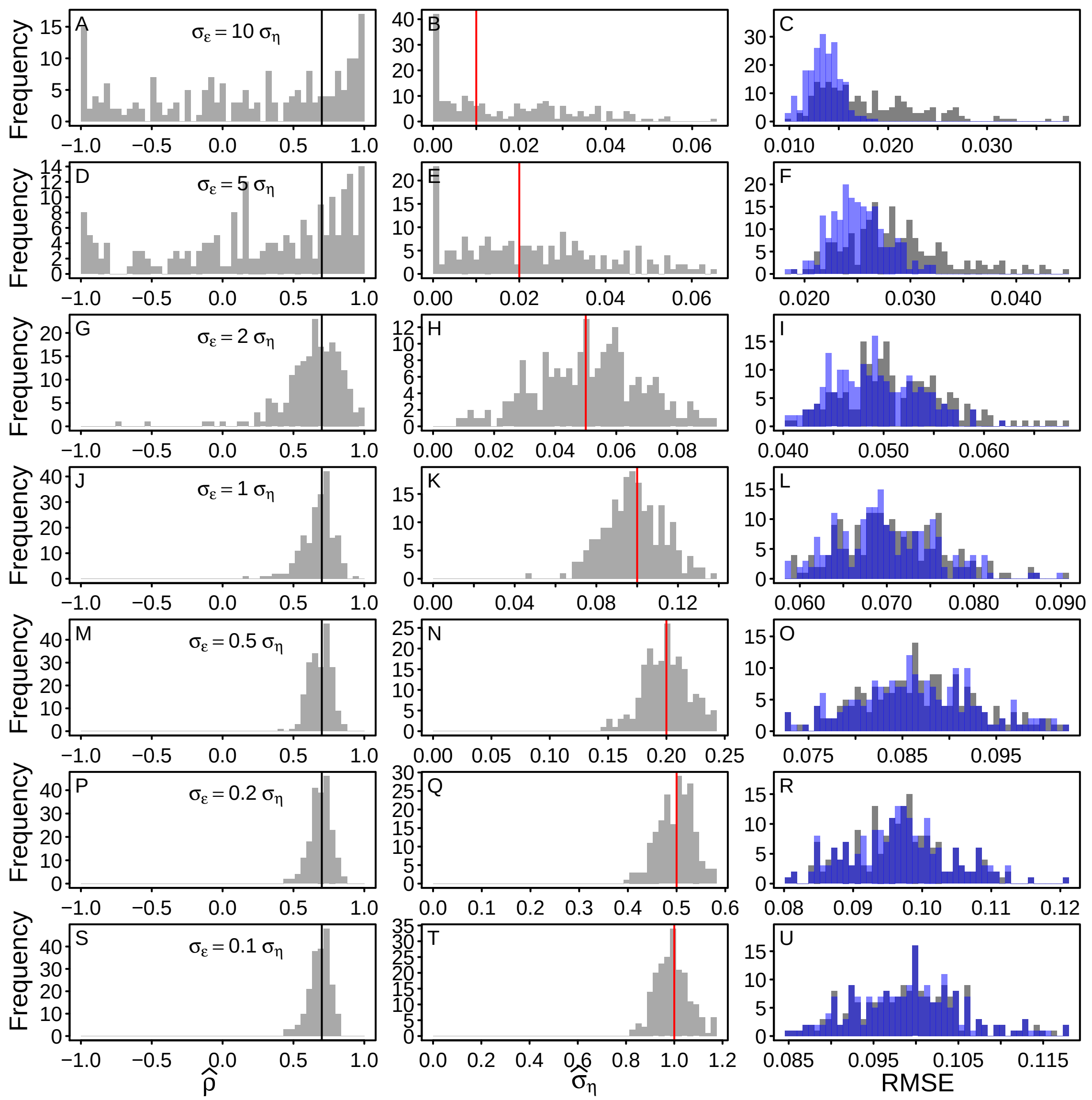

Figure G.1. Histograms of the parameter estimates and of the RMSE of the states when the values of the measurement error is fixed to its true value. Each row represents the results of 200 simulations for a set of parameter values. For the first three columns, the vertical lines represent the parameter values used in the simulations, with black lines used for the values that remain constant, $\sigma_\varepsilon = 0.1$ and $\rho = 0.7$, and the red lines for values that change between set, $\sigma_\eta = (0.01, 0.02, 0.05, 0.1, 0.2, 0.5, 1)$. In the last column, the grey histograms represent the RMSE of the model fitted using the estimated parameter values, while the blue histograms represent the RMSE when the model was fitted using the simulation values.

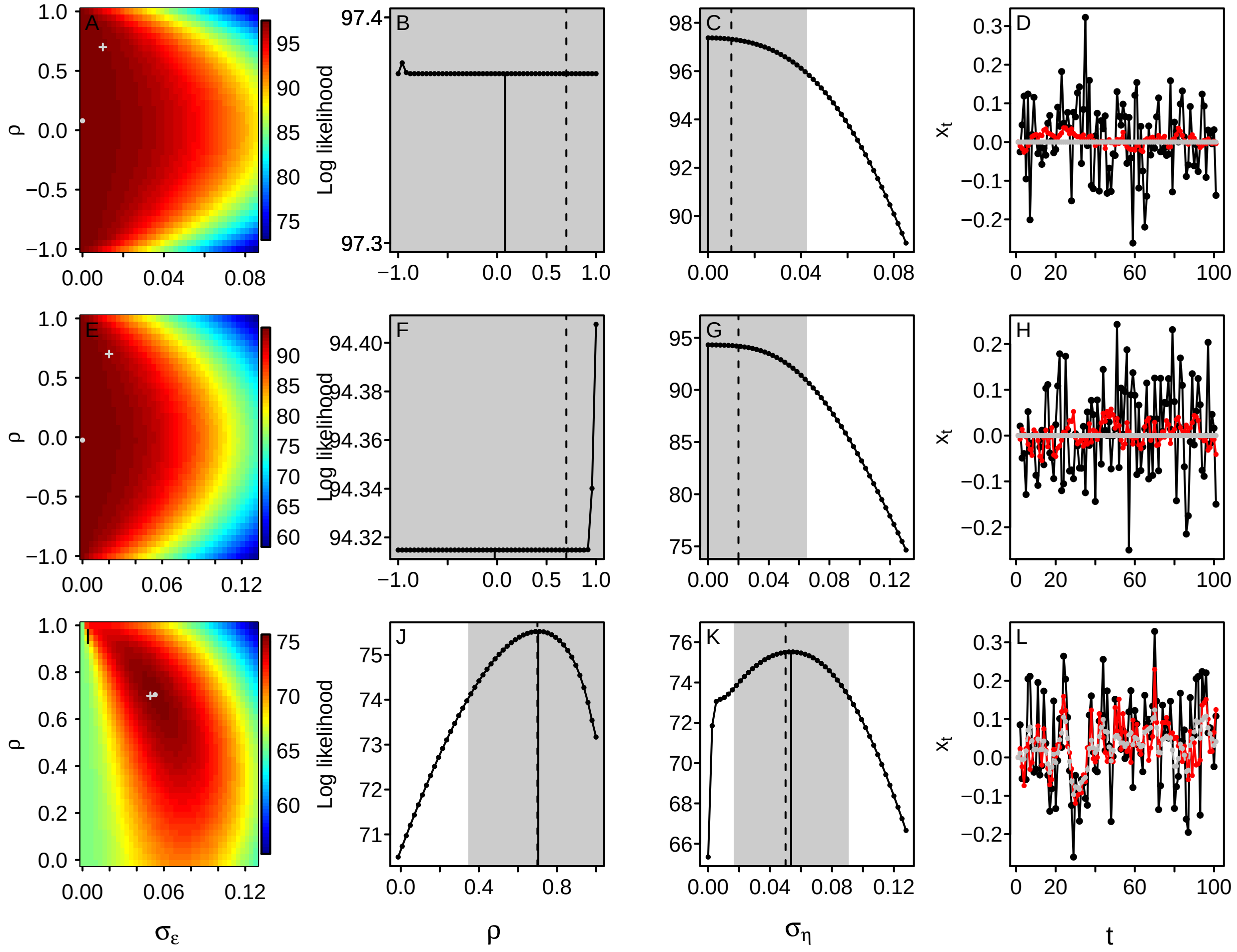

Figure G.2. Log likelihood surface and profile for the problematic simulations when the standard deviation of the measurement equation is fixed to the simulated value, $\sigma_\varepsilon = 0.1$. The first column represents the log likelihood surface for the two estimated parameters, $\theta_{\mathbf{m}} = (\rho, \sigma_\eta)$. The grey dot is the maximum likelihood estimate and the grey cross is the simulated value. The second and third columns represent the log likelihood profile for ρ and σ_η. The curve represents the log likelihood when the focal parameter is fixed (the other parameter are optimise to maximise the log likelihood). The dash lines are the true parameter values (i.e., value used for the simulation), the full lines are the maximum likelihood estimates and the grey bands represent the 95% CI. The last column shows the time-series. The black lines represent the observations, y_t, the red lines the simulated true states, x_t, and the grey dashed lines the estimated states, $\hat{x}_t$.

H Diagnostic tools

Identifying whether the model has parameter estimability problems is an important step towards avoiding biased inference. In this Appendix, we present a few diagnostic tools for both the more traditional likelihood-based methods and for Bayesian methods.

One of the best way to check whether a model is capable of estimating the parameters and states is through a simulation study such as the one presented in this manuscript. While an extensive simulation study that investigates a variety of parameter values is necessary to assess the overall capacity of the model, in many cases it may be sufficient to focus on parameter values similar to those estimated from the real data. An example of such focussed simulation study is presented in Appendix F. One important aspect of these simulation studies is that they require to run a large sample of simulations (we used a sample of 200-500 simulations per parameter set). Using a single simulation can be extremely misleading. For instance, only 29.6% of our main simulations were problematic (see section Simulations results from the main text). However, repeatedly fitting a model to multiple time-series may only be feasible with computationally-efficient method. Computing a simulation study with `rjags` is much less practical than with `TMB`.

As shown repeatedly in the manuscript, one way to identify the potential for estimation problems with likelihood-based methods is by investigating the profile likelihood. Flat, jagged, or bimodal profile likelihoods indicate the potential for parameter-estimation problems (e.g., Fig. 2A-C). In contrast, smooth unimodal profile likelihoods, such as those where we know the true states (e.g., Fig. D.1A-C), indicate that there is no obvious estimation problems. A more comprehensive investigation can be done through the visualization of a likelihood surface.[12] For example, by looking at the parameters two-by-two. As an example, we used one of the problematic simulations (i.e., $\mathrm{RMSE}_{\hat{\theta}} > 1.5 \times \mathrm{RMSE}_{\theta}$), where $\sigma_\eta = 0.05$. We computed the likelihood surface for the measurement error and process stochasticity. To demonstrate the difference between a problematic and a well-behaved model, we compared the case when the states were estimated (as in the main text) to the case when the states were known (Appendix D). We can see that when the states are estimated the likelihood surface has a diagonal ridge indicating that it is difficult to separate the values of σ_η from those of σ_ε (Fig. H.1A). In contrast, in the case when the states are known, we have a well-behaved unimodal likelihood surface (Fig. H.1B).

When the model is fitted with Bayesian methods, chain convergence is often used as a diagnostic. The sample paths of MCMC chains for non-identifiable parameters may interchange their values and lead to numerical or convergence problems.[13] However, in our case, very few replicates had convergence problems and many of the converged chains lead to biased estimates (see Appendix C). To further investigate the potential for estimation problems, in particular, to verify whether the estimates from the different parameters are correlated, one can investigate the posterior distribution of parameters. To show how this

method has similarity to investigating the likelihood surface, we used the same example as above. We compared the posterior distribution of model described in eq. 1-2 (see Appendix C for the description of priors) when the states were estimated as opposed to when the states were known. As we can see in Fig. H.2A, when the states are estimated the posterior distribution of the measurement error and process stochasticity appears strongly correlated, indicating that there is an estimation problem. In contrast, the posterior distribution when the states are known does not appear correlated (Fig. H.2B), indicating that there is no obvious estimation problem. This is consistent with the results of Appendix D, which shows that when we know the true states, the estimated parameters are close to their true values.

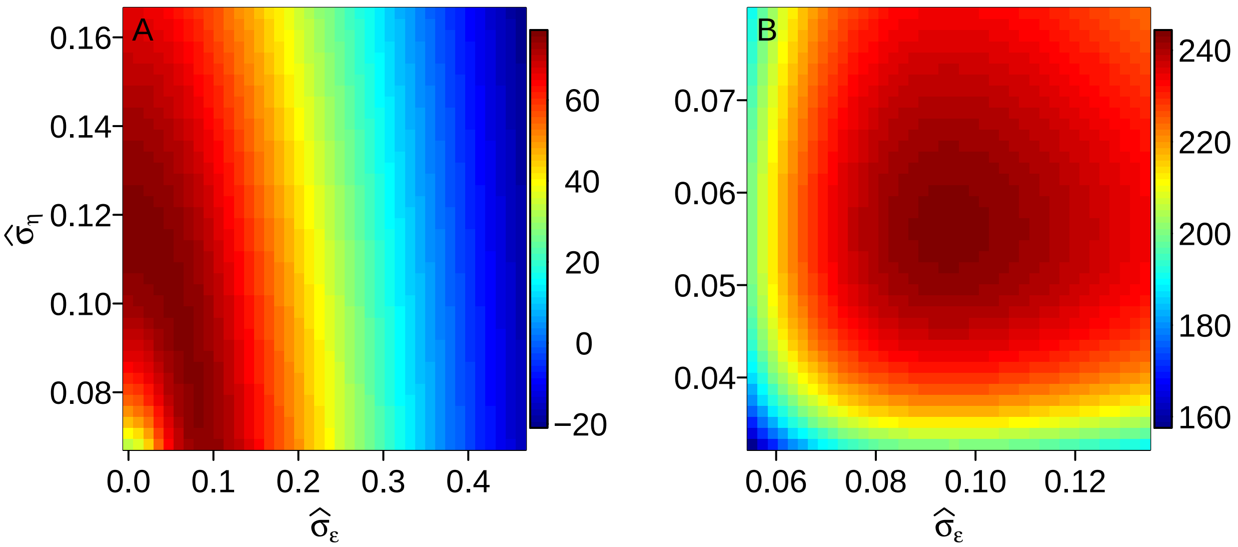

Figure H.1. Log likelihood surface for the measurement error and process stochasticity. A) Surface when the states and parameters are estimated. B) Surface when the true states are known.

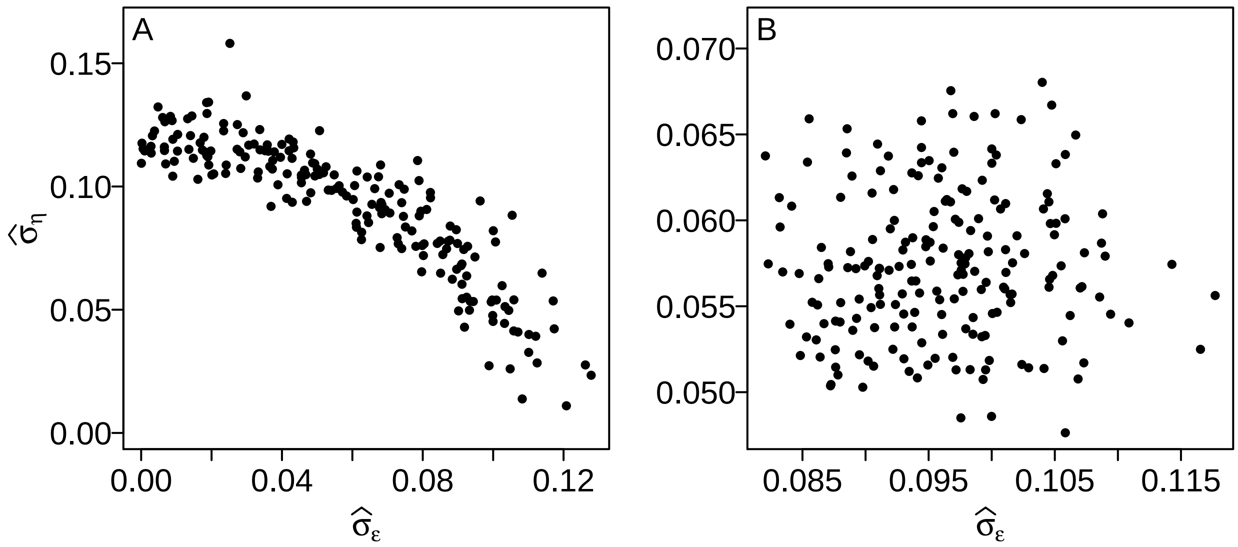

Figure H.2. Posterior distribution of the measurement error and process stochasticity. A) Distribution when the states and parameters are estimated. B) Distribution when the true states are known.

References

1. Knape, J. Estimability of density dependence in models of time series data. *Ecology* **89**, 2994–3000 (2008).
2. Forester, J. D. *et al.* State-space models link elk movement patterns to landscape characteristics in Yellowstone National Park. *Ecological Monographs* **77**, 285–299 (2007).
3. Petris, G. An R package for dynamic linear models. *Journal of Statistical Software* **36**, 1–16 (2010).
4. Durbin, J. & Koopman, S. J. *Time series analysis by state space methods* (Oxford University Press, Oxford, 2001).
5. Simmons, R. E., Kolberg, H., Braby, R. & Erni, B. Declines in migrant shorebird populations from a winter-quarter perspective. *Conservation Biology* **29**, 877–887 (2015).
6. Dennis, B., Ponciano, J. M., Lele, S. R., Taper, M. L. & Staples, D. F. Estimating density dependence, process noise, and observation error. *Ecological Monographs* **76**, 323–341 (2006).
7. Humbert, J.-Y., Mills, L. S., Horne, J. S. & Dennis, B. A better way to estimate population trends. *Oikos* **118**, 1940–1946 (2009).
8. Albertsen, C. M., Whoriskey, K., Yurkowski, D., Nielsen, A. & Mills Flemming, J. Fast fitting of non-Gaussian state-space models to animal movement data via Template Model Builder. *Ecology* **96**, 2598–2604 (2015).
9. Tittensor, D. P. *et al.* A mid-term analysis of progress toward international biodiversity targets. *Science* **346**, 241–244 (2014).
10. Plummer, M. rjags: Bayesian graphical models using MCMC. R package version 3-13. (2014).
11. Box, G. E., Jenkins, G. M. & Reinsel, G. C. *Time series analysis: forecasting and control* (Wiley, Hoboken, 2008), 4th edn.
12. Polansky, L., de Valpine, P., Lloyd-Smith, J. O. & Getz, W. M. Likelihood ridges and multimodality in population growth rate models. *Ecology* **90**, 2313–2320 (2009).
13. Cressie, N., Calder, C. A., Clark, J. S., Ver Hoef, J. M. & Wikle, C. K. Accounting for uncertainty in ecological analysis: the strengths and limitations of hierarchical statistical modeling. *Ecological Applications* **19**, 553–570 (2009).
